# Intrauterine hyperglycemia exposure results in intergenerational inheritance via DNA methylation reprogramming on F1 PGCs

**DOI:** 10.1186/s13072-018-0192-2

**Published:** 2018-05-25

**Authors:** Jun Ren, Yi Cheng, Zhen-Hua Ming, Xin-Yan Dong, Yu-Zhong Zhou, Guo-Lian Ding, Hai-Yan Pang, Tanzil Ur Rahman, Rubab Akbar, He-Feng Huang, Jian-Zhong Sheng

**Affiliations:** 1 Department of Pathology and Pathophysiology, School of Medicine, Zhejiang University, Hangzhou, China; 2 The Key Laboratory of Reproductive Genetics (Zhejiang University), Ministry of Education, Hangzhou, China; 3 Department of Reproductive Endocrinology, Zhejiang Women’s Hospital, School of Medicine, Zhejiang University, Hangzhou, China; 4 The International Peace Maternity and Child Health Hospital, School of Medicine, Shanghai Jiao Tong University, Shanghai, China

**Keywords:** DNA methylation, Intrauterine hyperglycemia, Primordial germ cells, Reduced representation bisulfite sequencing, Epigenetic inheritance

## Abstract

**Background:**

The existing reports about intergenerational or transgenerational effects of intrauterine hyperglycemia have included both intrauterine and postnatal metabolic exposure factors, while the impact of intrauterine hyperglycemia per se has not been assessed alone. A number of studies suggest DNA methylation reprogramming of gametes plays a crucial role in the metabolic inheritance, but it is unclear when and how DNA methylation patterns are altered when exposed to intrauterine hyperglycemia. In this study, we selected nondiabetic F1- and F2-gestational diabetes mellitus (GDM) male mice as founders to examine metabolic changes in the next generation and performed methylome sequencing of day 13.5 primordial germ cells (PGCs) from F1-GDM to explore the underlying epigenetic mechanism.

**Results:**

We found that intrauterine hyperglycemia exposure resulted in obesity, insulin resistance, and/or glucose intolerance in F2 male mice, but no metabolic changes in F3 male mice at 8 weeks. Using reduced representation bisulfite sequencing, we found DNA methylome of day 13.5 PGCs from F1-GDM fetuses revealed differently methylated genes enriched in obesity and diabetes. Methylation validation of the insulin resistance and fat accumulation gene *Fyn* showed a consistent hypomethylation status in F1 PGCs, F1 fetal testes, sperm from F1/C-GDM mice, and somatic cells from F2-GDM male mice. In contrast, no methylation alteration was observed in F2-GDM male germ cells and F3-GDM somatic cells.

**Conclusion:**

We provide evidence that intrauterine hyperglycemia exposure per se contributes to intergenerational metabolic changes in the F2 but not F3 generation. And the aberrant DNA methylation reprogramming occurs as early as day 13.5 in PGCs of the F1 generation. Our findings suggest that intrauterine exposure alone is sufficient to cause the epigenetic inheritance in F2 offspring, and the epigenetic memory carried by DNA methylation pattern could be erased by the second wave of methylation reprogramming in F2 PGCs during fetal development.

**Electronic supplementary material:**

The online version of this article (10.1186/s13072-018-0192-2) contains supplementary material, which is available to authorized users.

## Background

The concept of “developmental origins of health and disease (DOHaD)”, which states that an adverse intrauterine environment during critical periods in early life may lead to disease risk in later life, is now widely accepted [1]. This theory has been supported by abundant results from both human epidemiological studies and animal models [2–6]. Adverse factors, such as imbalanced nutrition intake, smoking, caffeine, bisphenol A, and drug use, affect multiple aspects of offspring health [2–6]. Accumulating evidence also suggests that adverse intrauterine exposure may not only directly influence the development of the fetus (F1 offspring), but also the germ cells of F1 that will form the F2 generation [7].

Gestational diabetes mellitus (GDM) is defined as glucose intolerance and hyperglycemia with first onset during pregnancy [8]. GDM is the most common complication of pregnancy, affecting 5–10% of all pregnancies [9]. Children born to mothers with GDM are prone to develop chronic diseases such as obesity, impaired glucose tolerance, cardiovascular disorders, and type II diabetes in adulthood [10–13]. In addition, our previous study suggested that the second generation might exhibit similar metabolic dysfunction phenotypes to their parents, especially by paternal line [14]; however, in this study sperm of the F1 generation underwent two major phases of adverse environment exposure: prenatal exposure to intrauterine hyperglycemia and postnatal metabolic dysfunction to the F1 generation itself. It has been demonstrated that postnatal induced-diabetic conditions in healthy male mice could alter the epigenetic information in sperm, and these epigenetic changes were largely heritable [15]. The offspring showed diabetic phenotypes that resembled their fathers [15]. Up to now, it is unclear whether intrauterine hyperglycemia exposure itself may affect epigenome of gamete precursors of the F1 fetus. Therefore, it is necessary to investigate the effects of intrauterine hyperglycemia on epigenetic alteration in primordial germ cells (PGCs) of the F1 fetus and assess whether and how intrauterine hyperglycemia results in metabolic changes in the F2 generation.

The term transgenerational inheritance is broadly used to describe all nonsequence-based effects that can be transmitted to next generation. However, it is important to distinguish transgenerational effects from intergenerational effects. Truly transgenerational effect is found in generations that were not exposed to the initial environmental exposure that trigger the change. A nexus of mechanisms may contribute to the transmission effect, including self-sustaining feedback loops, chromatin-based mechanisms, noncoding and coding RNA, even nonepigenetic mechanisms like microbiotic effects or metabolites [16]. DNA methylation is the best studied chromatin-based mechanism in this field.

DNA methylation is a major form of epigenetic modification; it is crucial for the development and differentiation of various cell types in an organism. Substantial evidence has demonstrated that altering the DNA methylation pattern contributes to the inheritance of acquired characteristics [17–20]. Two waves of genome-wide DNA methylation reprogramming occur during mouse development. The first wave of reprogramming occurs in pre-implantation blastocyst embryos, and the second wave occurs solely in PGCs [21]. PGCs are specified in mice during gastrulation. The second wave of global DNA methylation modification of PGCs is erased at day (D) 12.5–13.5 in mice after colonization into the genital ridges [22, 23] and then re-established when PGCs differentiate into the precursors of gametes at D18.5 [24].

Previous studies that investigated the mechanisms underlying the intergenerational effects of GDM have usually focused on mature germ cells rather than PGCs. Therefore, it has not yet been studied whether exposure to intrauterine hyperglycemia affects the DNA methylation reprogramming of PGCs. In this study, we built a GDM mouse model and selected male F1- and F2-GDM mice without the pre-diabetic phenotype as founders to produce the next generation. Thereby we investigated the direct effects of intrauterine hyperglycemia exposure on the F2 and F3 generations. We also assessed DNA methylation reprogramming in male PGCs from F1 fetuses, and its heritability to germ cells and somatic cells in F2 and F3 generation.

## Methods

### Establishing the GDM mouse model

The protocol was approved by the Zhejiang University Institutional Animal Care and Use Committee. The animals were housed in a temperature- and humidity-controlled environment under a 12-h light/dark cycle. Eight-week-old normal virgin female ICR mice were mated with normal males overnight. The onset of pregnancy was determined by the presence of a vaginal plug the following morning and defined as D0.5 of gestation. Gestational mice (F0) were randomly divided into control and intrauterine hyperglycemia (GDM) groups. After an 8-h fast, mice in the GDM group were treated with a single intraperitoneal injection of streptozotocin (STZ; Sigma, St. Louis, MO, USA) in 0.1 mM citrate buffer (pH 4.5) at a dose of 150 mg/kg bodyweight. Mice in the control group received an equal volume of citrate buffer. On D3.5 of pregnancy, the blood glucose concentration was measured via the tail vein using a glucometer (Roche Diagnostics Accu-Chek, Mannheim, Germany). Diabetes was defined as a glucose level between 14 and 19 mM [25, 26]. Blood glucose levels were also monitored on D7.5 of pregnancy to confirm the presence of diabetes. The pregnant mice were allowed to deliver spontaneously. Pups born to the GDM mother (F1-GDM) were fostered by normoglycemic control mothers until they were weaned at 3 weeks of age. All mice were fed a standard diet.

F1-GDM male mice without metabolic phenotypes were collected as F1/C-GDM. F1/C-GDM mice were mated with control females to produce F2-GDM. Similarly, F2-GDM male mice without metabolic phenotypes (F2/C-GDM) were mated with control females to produce F3-GDM. In our experiments, one GDM mother was capable of producing both GDM and C/GDM offspring. Only male pups were investigated.

### Glucose tolerance test (GTT) and insulin tolerance test (ITT)

For GTT, animals were fasted for 12 h before the test. An intraperitoneal injection of glucose (2 g/kg body weight) was given to each animal, and glucose levels were measured at 0, 30, 60, and 120 min after injection. The area under the curve (AUC) for the GTT was calculated. For ITT, an intraperitoneal injection of insulin (0.8 U/kg body weight) was performed after a 3-h fast. Blood glucose concentrations were measured from the tail vein at 0, 30, 60, 90, and 120 min after injection. The AUC was also calculated for the ITT.

### Serum biochemical measurements

Blood samples were collected from 8-week-old mice after a 12-h fast. The serum levels of fasting insulin were determined with the mouse insulin enzyme-linked immunosorbent assay (ELISA) kit (Crystal Chem, Downers Grove, IL, USA). Serum triglyceride (TG), total cholesterol (TC), high-density lipoprotein (HDL), and low-density lipoprotein (LDL) were assayed using a biochemical analyzer (TBA120FR, Toshiba, Tokyo, Japan). Fasting glucose levels were measured using a glucometer (Roche Diagnostics Accu-Chek). The Homeostasis Model of Insulin Resistance (HOMA-IR) index was calculated using the formula: HOMA-IR = (fasting insulin × fasting glucose)/22.5.

### Reduced representation bisulfite sequencing (RRBS) and data analysis

RRBS (Genergy Biotechnology Co., Ltd., Shanghai, China) was performed in PGCs of D13.5 male fetuses from control pregnant mice (F0-control, *n* = 8) and GDM pregnant mice (F0-GDM, *n* = 8). PGCs were pooled from two pregnant mice to obtain the amount of material needed for RRBS. Briefly, 5 μg genomic DNA was digested using the methylation-insensitive restriction enzyme *Msp*I (New England Biolabs, Beverly, MA, USA). A Qiagen Mini Purification kit (Qiagen, Hilden, Germany) was used to purify the digested products. Then, the ends of each restriction fragment were filled in and adenosine was added at the 3′-end. Methylated paired-end Illumina adapters were ligated to the ends of the DNA fragments using T4 DNA ligase, and fragments sized 100–200 bp were purified by agarose gel extraction. The purified fragments were treated with sodium bisulfite and then amplified by PCR. The final PCR products were sequenced on HiSeq 2500 (Illumina Inc., San Diego, CA, USA). Differentially methylated loci (DML) and differentially methylated regions (DMRs) were analyzed based on a Bayesian approach [27], summarized as follows: two groups were modeled according to the Bayesian stratification model, and the Wald test was applied to each locus to get a p value for each CpG site. For each CpG site, a difference in methylation value between two groups ≥ 5% and a posteriori probability of Wald test ≥ 0.95 was considered to be a DML. A methylation region was defined as a DMR when it met these three criteria: (1) the length of this region was at least 50 bp; (2) the region contained no less than three CpG sites; (3) the proportion of DMLs in this region was no less than 50%. When a DMR showed no less than 50% overlap with one element of the gene, it was defined as a differentially methylated gene (DMG). Bioinformatic analysis of DMGs was performed using the Ingenuity Pathway Analysis (IPA) software package (Ingenuity Systems, Redwood City, CA, USA). The RRBS data reported in this paper have been deposited in the NCBI Gene Expression Omnibus (GEO) database with accession number GSE108319.

### DNA extraction and pyrosequencing PCR

DNA was isolated using TIANamp Genomic DNA Kit (Tiangen Biotech, Beijing, China). DNA bisulfite conversion was modified using EZ DNA CT conversion reagent (Zymo Research Corporation, Irvine, CA, USA) following the manufacturer’s protocol. The primers for *Fyn* bisulfite DNA PCR and sequencing were designed using Pyromark Assay Design 2.0 software (Biotage, Uppsala, Sweden) and listed in Additional file 1. Gene model of *Fyn* was presented as Additional file 1: Fig. S1.

Bisulfite DNA PCR was performed in a total volume of 25 μl using the TaKaRa EpiTaq HS system (TaKaRa, Dalian, China). Pyrosequencing was performed on a PyroMark Q24 MD system (Qiagen) using the PyroMark Gold Q24 reagent kit (Qiagen) with 10 pmol of sequencing primer. Data were analyzed using Pyro Q-CpG software (Qiagen).

### RNA isolation and quantitative real time-PCR (qPCR) analysis

RNA was extracted using TRIzol reagent (Life Technologies, Grand Island, NY, USA) and cDNA was synthesized using oligo-dT and random primers (TaKaRa). qPCR was performed using the ABI Prism 7900HT sequence detection system (Applied Biosystems, Foster City, CA, USA) with commercial primers generated for the system. *β*-*actin* was used as the internal control. Primers were listed in Additional file 1.

### Statistical analysis

SPSS Statistics 16 (SPSS Inc., Chicago, IL, USA) was used for all statistical analyses. Data are presented as mean ± SEM. Comparisons between two groups were performed using Student’s *t* tests. One-way ANOVA was used to detect differences among multiple groups followed by LSD post hoc test for comparing groups. All reported *p* values are two-sided. *p *< 0.05 was considered statistically significant.

## Results

### F1/C-GDM male mice had normal glucose and lipid metabolism

**Fig. 1:**
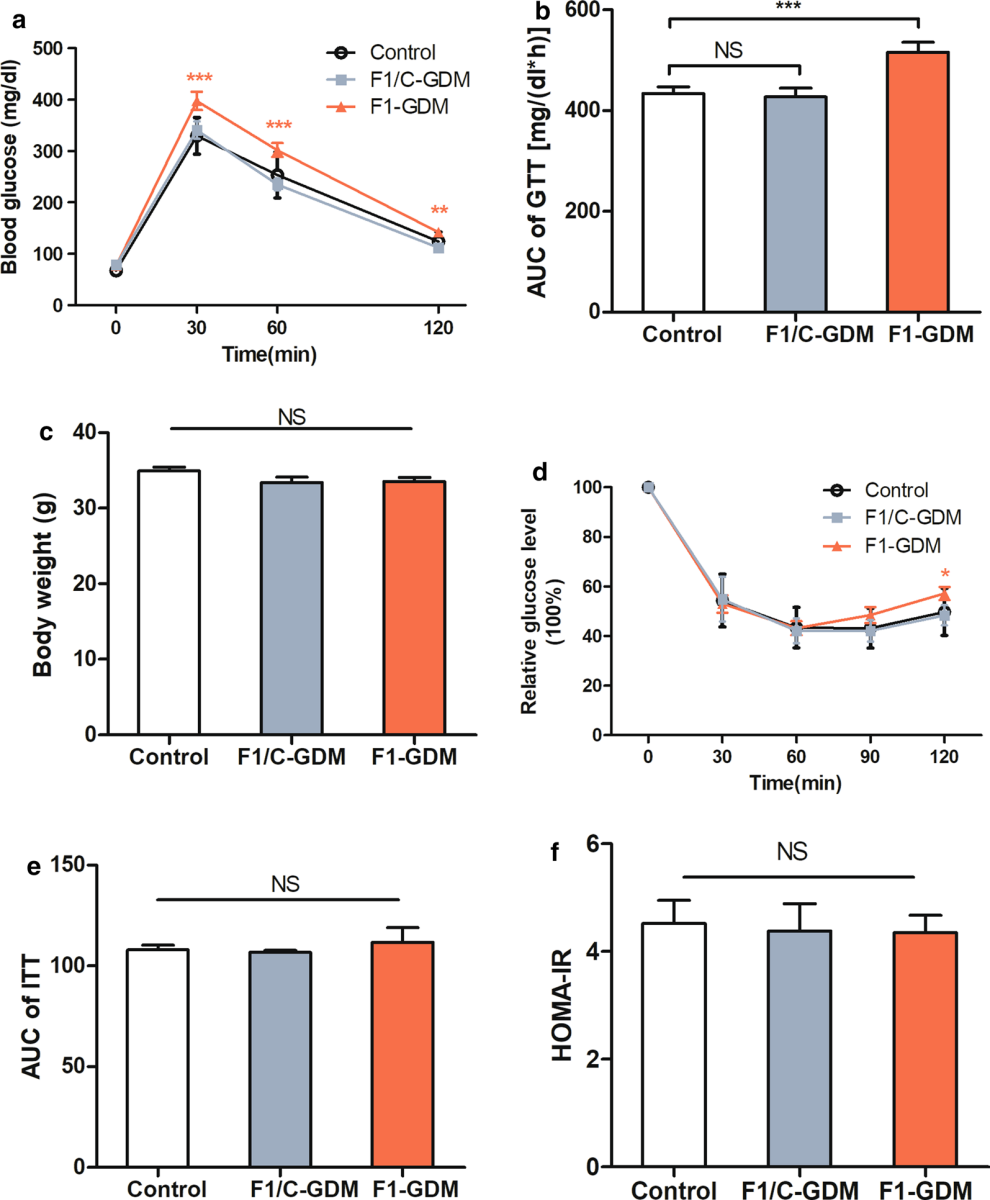
F1/C-GDM mice demonstrated an unaffected metabolic phenotype at adulthood. **a** GTT curve and **b** the resulting AUC of control, F1-GDM and F1/C-GDM male mice at 8 weeks. **c** Bodyweight of control, F1-GDM, and F1/C-GDM male mice at 8 weeks. **d** ITT curve and **e** the resulting AUC of control, F1-GDM, and F1/C-GDM male mice at 8 weeks. For each group in **a**–**e**, control (*n* = 10), F1-GDM (*n* = 25), and F1/C-GDM (*n* = 9). **f** HOMA-IR index of control (*n* = 10), F1-GDM (*n* = 10), and F1/C-GDM (*n* = 4) male mice at 8 weeks. Data are presented as mean ± SEM. *p < 0.05, **p < 0.01, ***p < 0.001 versus the corresponding control

GTT is the most commonly used method for assessing glucose homeostasis, and it provides a physiological overview of any changes in glucose tolerance. ITT and HOMA-IR index are used to measure insulin resistance of peripheral tissues. These three methods along with basal hormone levels provide a systemic view of glucose homeostasis. GTT and ITT tests showed that, compared with control male mice, 8-week-old F1-GDM male mice exhibited significant glucose intolerance (Fig. 1a, b). Though F1-GDM mice showed a significantly higher glucose level at 120 min in ITT, there was no difference in AUC of ITT (Fig. 1d, e) or HOMA-IR index (Fig. 1f) between the two groups. Further analysis showed that 36% (9/25) of the F1-GDM male offspring had normal glucose tolerance and insulin tolerance (Fig. 1b, e). These mice were separated and defined as GDM offspring without impaired glucose tolerance and impaired insulin tolerance (F1/C-GDM). There were no significant differences in bodyweight (Fig. 1c), fasting glucose level, fasting insulin level, TG, TC, HDL, LDL (Table 1), and HOMA-IR index (Fig. 1f) between the control and F1/C-GDM groups.

**Table 1:** Serum biochemical parameters in F1/C-, F2-, and F3-GDM male mice at 8 weeks

	Control (*n* = 10)	F1/C-GDM (*n* = 9)	F2/H-GDM (*n* = 10)	F2/N-GDM (*n* = 6)	F3-GDM (*n* = 10)
Fasting glucose (mM)	4.57 ± 0.23	4.38 ± 0.24	4.78 ± 0.20	4.54 ± 0.02	4.61 ± 0.31
Fasting insulin (mIU/l)	22.05 ± 6.97	21.87 ± 4.50	27.81 ± 9.95^##^	73.12 ± 9.45^***^	19.10 ± 7.43
TC (mM)	2.47 ± 0.06	2.65 ± 0.11	2.48 ± 0.11	2.48 ± 0.09	2.29 ± 0.16
TG (mM)	1.33 ± 0.11	1.32 ± 0.17	1.83 ± 0.12^*,#^	1.20 ± 0.14	1.24 ± 0.61
HDL (mM)	1.39 ± 0.03	1.54 ± 0.06	1.43 ± 0.05	1.37 ± 0.04	1.60 ± 0.10
LDL (mM)	0.20 ± 0.01	0.20 ± 0.01	0.16 ± 0.01^*^	0.18 ± 0.02	0.22 ± 0.01

*TC* total cholesterol; *TG* triacylglycerol; *HDL* high-density lipoprotein; *LDL* low-density lipoprotein. All parameters were measured at 8 weeks of age. Values are expressed as mean ± SEM

*p < 0.05; ***p < 0.001 compared with the control group

^#^*p *< 0.05; ^##^*p *< 0.01 compared with the F2/N-GDM group

### Adult F2-GDM male mice from F1/C-GDM fathers exhibited obesity, insulin resistance, and/or glucose intolerance

**Fig. 2:**
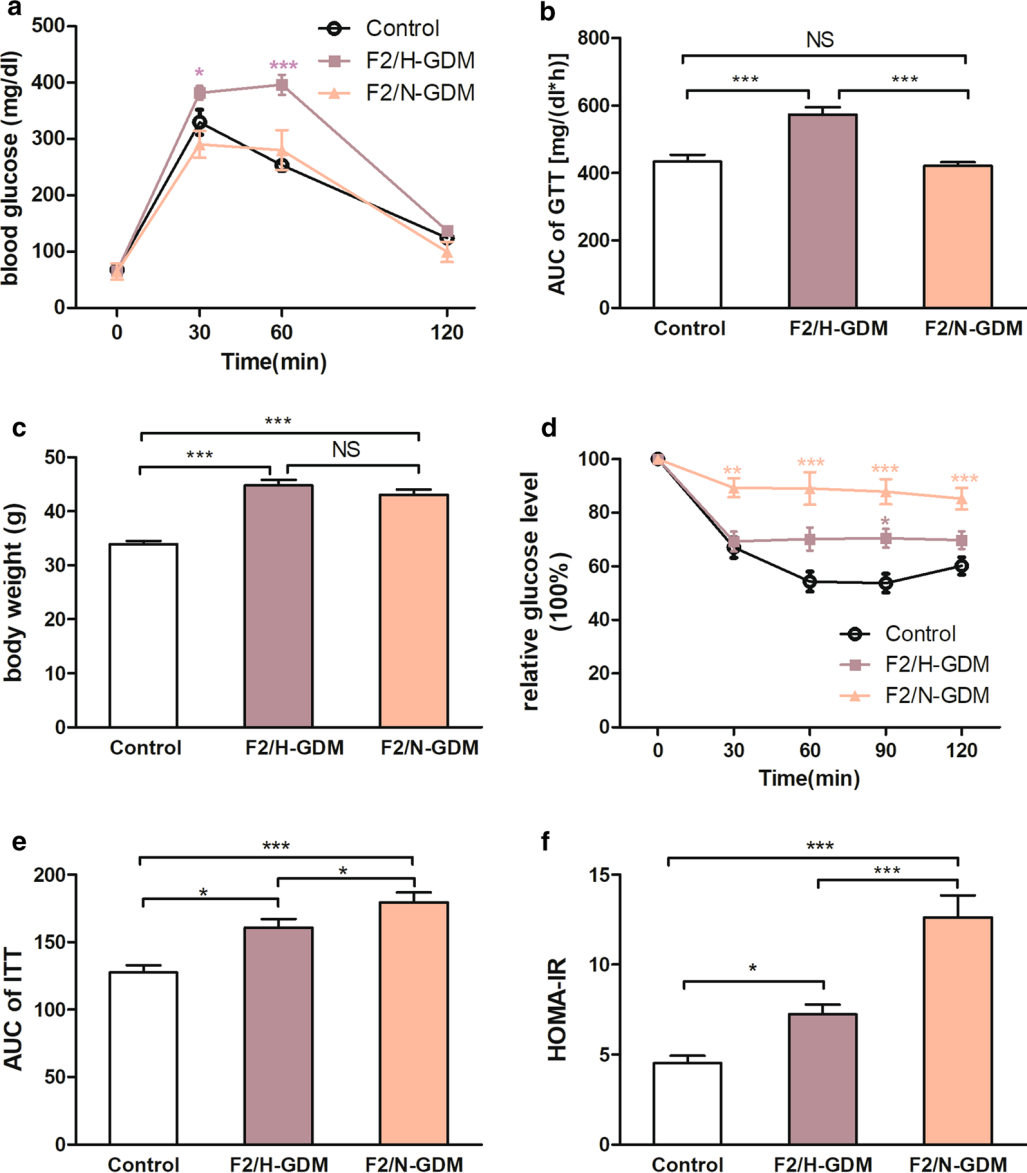
F2-GDM mice demonstrated an impaired metabolic phenotype at adulthood. **a** GTT curve and **b** the resulting AUC in control, F2/H-GDM, and F2/N-GDM male mice at 8 weeks. **c** Bodyweight in control, F2/H-GDM, and F2/N-GDM male mice at 8 weeks. **d** ITT curve and **e** the resulting AUC of control, F2/H-GDM, and F2/N-GDM male mice at 8 weeks. For each group in **a**–**e**, control (*n* = 10), F2/H-GDM (*n* = 10), and F2/N-GDM (*n* = 6). **f** HOMA-IR index of control (*n* = 10), F2/H-GDM (*n* = 10), and F2/N-GDM (*n* = 6) male mice at 8 weeks. Data are presented as mean ± SEM. *p < 0.05, **p < 0.01, ***p < 0.001 versus the corresponding control

F1/C-GDM male mice were mated with control female virgin to produce second-generation mice, named F2-GDM. GTT was conducted at 8 weeks. Then, the F2-GDM mice were divided into two groups according to the AUC of the GTT: F2/H-GDM (with impaired glucose tolerance) and F2/N-GDM (with normal glucose tolerance). Glucose levels were significantly higher at 30 and 60 min in F2/H-GDM mice compared with the control group (*p *< 0.05 and *p *< 0.001, respectively; Fig. 2a). The AUC of F2/H-GDM mice was significantly larger than that of control and F2/N-GDM mice (*p *< 0.001; Fig. 2b). There was no difference in GTT between the F2/N-GDM and control groups (Fig. 2a, b).

At 8 weeks of age, the mean bodyweight of mice in the F2/H-GDM and F2/N-GDM groups was significantly higher than that of control mice (*p *< 0.001 and *p *< 0.001, respectively; Fig. 2c). There was no significant difference in bodyweight between F2/H-GDM and F2/N-GDM mice (Fig. 2c).

ITT was performed to assess insulin sensitivity of the F2 generation. Although glucose levels were only increased in F2/H-GDM mice at 90 min (*p *< 0.05), the AUC of the ITT was increased significantly (*p *< 0.05) compared with control mice. The F2/N-GDM group exhibited continuously higher glucose levels at 30, 60, 90, and 120 min (*p *< 0.01, *p *< 0.001, *p *< 0.001, and *p *< 0.001, respectively), and the AUC was significantly larger than that of the control group (*p *< 0.001; Fig. 2d, e).

HOMA-IR demonstrated the same trend as the ITT results in the F2 generation. There were no differences in fasting glucose levels among the three groups. Fasting insulin was higher in the F2/H-GDM group than the control group, but not significantly (Table 1). In the F2/N-GDM group, fasting insulin levels were significantly increased compared with the control and F2/H-GDM groups (*p *< 0.001 and *p *< 0.01, respectively; Table 1). HOMA-IR was significantly higher in the F2/H-GDM group than the control group (*p *< 0.05; Fig. 2f). In addition, HOMA-IR was significantly higher in the F2/N-GDM group than the control and F2/H-GDM groups (*p *< 0.001 and *p *< 0.01, respectively; Fig. 2f). There were no differences in serum TC, TG, HDL, and LDL levels between F2/N-GDM and control mice (Table 1). In contrast, F2/H-GDM mice exhibited elevated TG and reduced LDL levels compared with control (*p *< 0.05, and *p *< 0.05, respectively; Table 1). Finally, TG levels were significantly higher in F2/H-GDM mice compared with F2/N-GDM (*p *< 0.05; Table 1). Several F2-GDM mice without any abnormal phenotype were selected as F2/C-GDM mice to produce F3 generation (Additional file 2: Table S1, Fig. S2). The proportion of F2/C-GDM mice was 15% (6/40 in F2-GDM male mice), less than that in F1/C-GDM.

### The methylome of D13.5 PGCs in F1 male fetus was altered by intrauterine hyperglycemia exposure

In our F1/C-GDM mouse model, exposure to hyperglycemia occurred only during pregnancy with no exposure after birth. However, this exposure resulted in next-generation obesity, insulin resistance, and/or glucose intolerance. This suggests that altered heritable information carried by gametes, such as epigenetic DNA methylation, is responsible for this susceptibility. We therefore used genome methylation sequencing to search for differentially methylated loci between control and F1-GDM fetuses. PGCs undergo global methylation erasure at D13.5 during pregnancy [28], and the methylation status was relatively stable [21]. We hypothesized that early epigenetic reprogramming in PGCs was altered during intrauterine hyperglycemia exposure. To test this hypothesis, we selected D13.5 PGCs from control and GDM fetuses and performed RRBS.

The gender of the D13.5 PGCs was identified under an anatomical lens and confirmed by *Sry* PCR results (Additional file 1: Fig. S3). Intrauterine hyperglycemia exposure resulted in 19,679 DML that were distributed in the upstream 2 k (2.79%), 5′-untranslated region (5′-UTR, 1.1%), coding sequence (CDS, 16.02%), introns (33.48%), 3′-UTR (2.29%), downstream 2 k (2.7%), and other elements (45.21%) of genes (Additional file 3: Fig. S4–S5). The 19,679 DML were associated with 304 genes that were then used in IPA analysis (Additional file 4: Table S2).

**Fig. 3:**
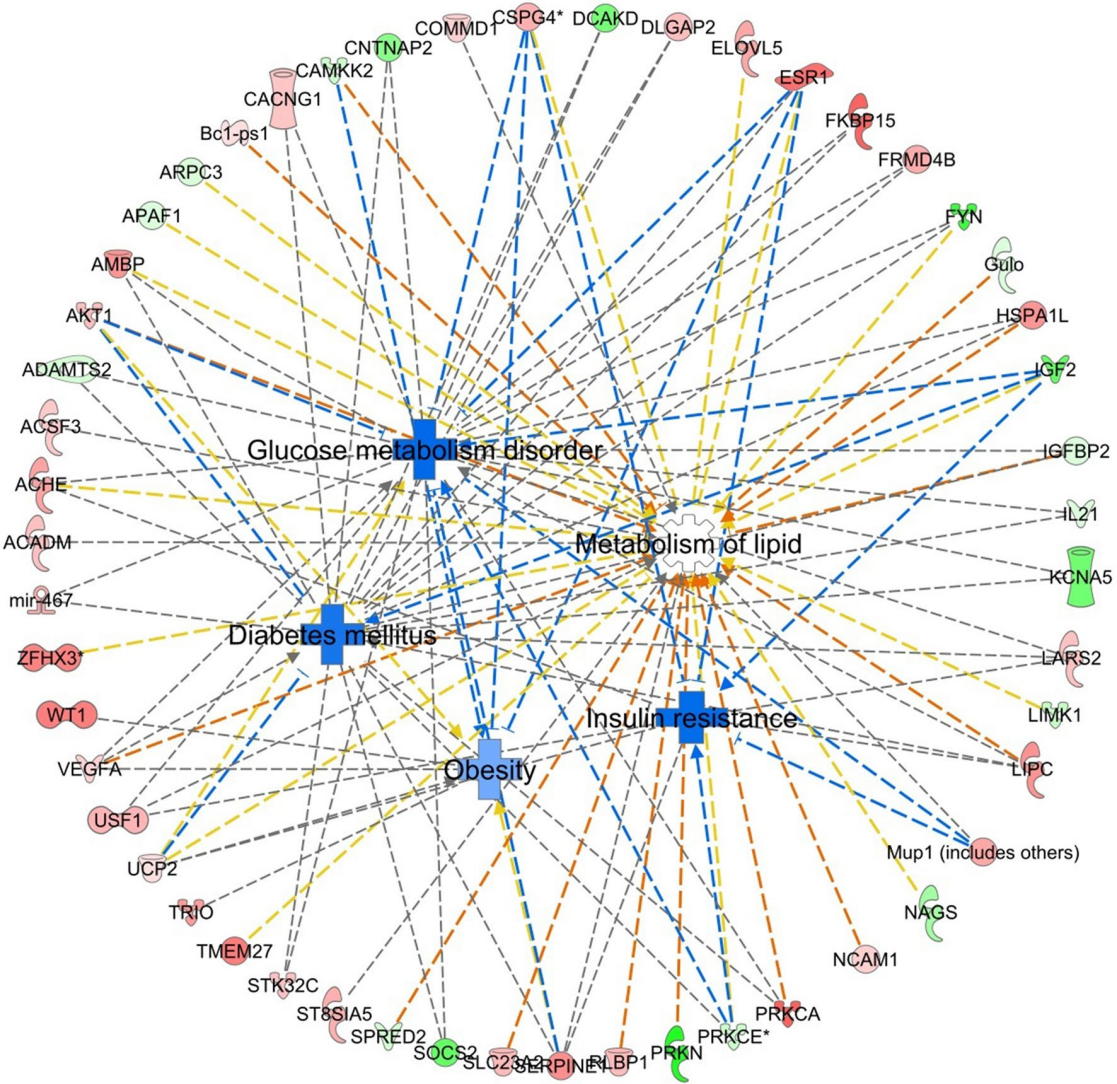
IPA analysis of reduced representation bisulfite sequencing in D13.5 PGCs from F1-GDM male fetuses. DMGs enriched in glucose metabolism disorder, lipid metabolism disorder, obesity, insulin resistance, and diabetes mellitus. Network analysis was performed using Ingenuity Pathway Analysis. Red color indicates hypermethylation, green color indicates hypomethylation, a solid line indicates a direct interaction, a dashed line indicates an indirect interaction, and a line without an arrowhead indicates binding

**Fig. 4:**
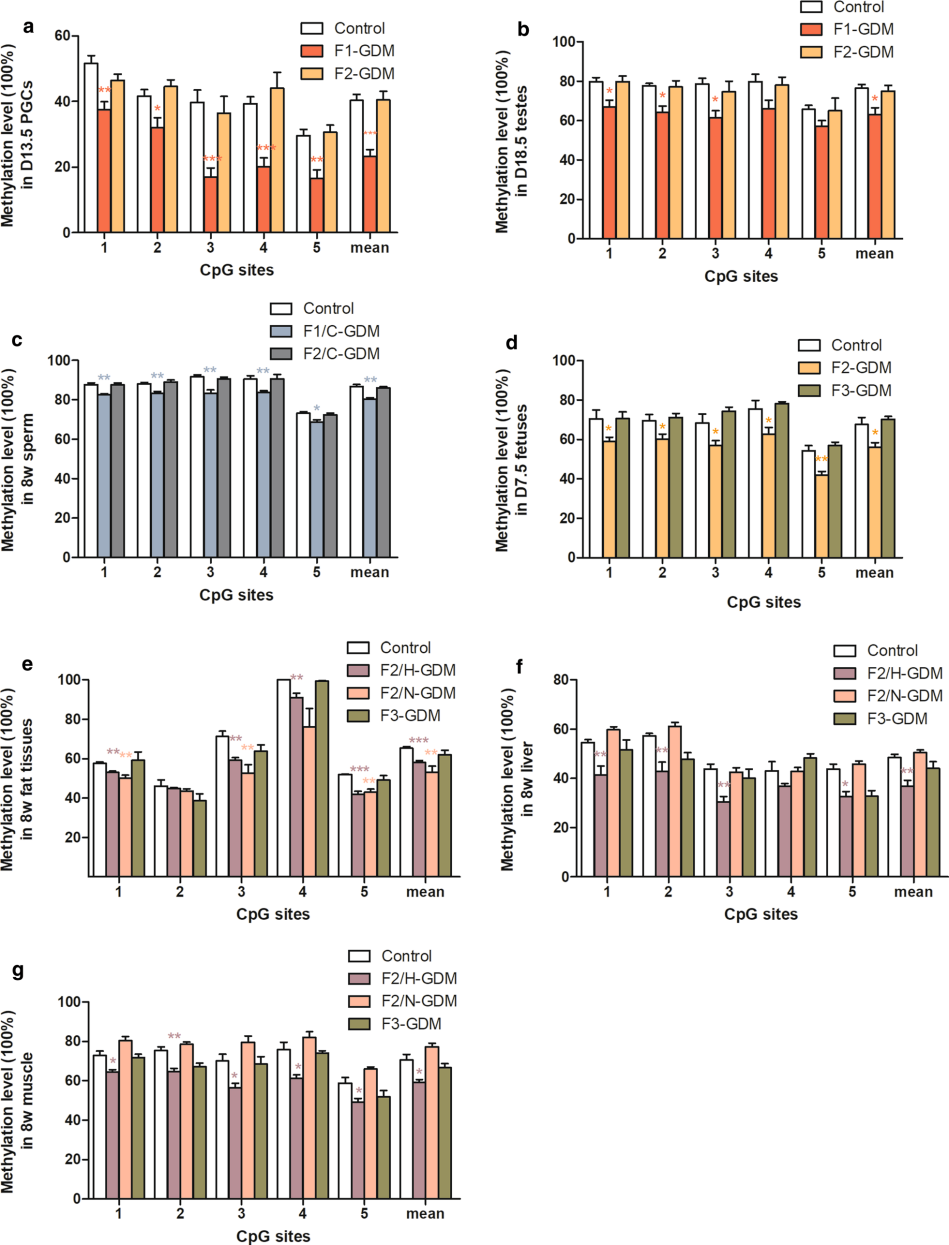
Targeted bisulfite pyrosequencing-based DNA methylation differences of *Fyn* in control, F1-, F2-, and F3-GDM male mice. **a** Methylation differences in D13.5 PGCs from control and GDM male fetuses (*n* = 5 pregnant mice per group). **b** Methylation differences in D18.5 control, F1-, and F2-GDM testes (*n* = 8 mice per group). **c** Methylation differences in sperm from control, F1/C-GDM, and F2/C-GDM mice at 8 weeks (*n* = 5 mice per group). **d** Methylation differences in D7.5 fetuses from control, F2-, and F3-GDM mice (*n* = 5 mice per group). **e** Methylation differences in adipose tissues from control, F2/H-GDM, and F2/N-GDM male mice at 8 weeks (*n* = 5 mice per group). **f** Methylation differences in liver tissues from control, F2/H-GDM, and F2/N-GDM male mice at 8 weeks (*n* = 5 mice per group). **g** Methylation differences in muscle tissues from control, F2/H-GDM, and F2/N-GDM male mice at 8 weeks (*n* = 5 mice per group). Data are presented as mean ± SEM. *p < 0.05, **p < 0.01, ***p < 0.001 versus the corresponding control

IPA analysis identified a cluster of differentially methylated genes that were strongly related to diabetes mellitus, glucose metabolism, lipid metabolism, obesity, and insulin resistance (Fig. 3), including hypermethylated genes such as *Akt1,* which regulates glucose uptake into muscle and fat cells [29]; *Elovl5*, which regulates blood glucose [30]; *Esr1,* which is involved in lipid accumulation and insulin sensitivity [31]; *Prkca*, which is involved in insulin signaling [32], and hypomethylated genes such as *Socs2*, which leads to hyperglycemia and glucose intolerance [33]; *Fyn*, which is essential in fat accumulation and insulin resistance [34]; *Park2*, which targets to mitochondria and potentiates autophagic vesicle synthesis [35]. Pathway details of the IPA analysis (Table S3) were attached as Additional file 5. Further, we performed targeted bisulfite pyrosequencing of *Fyn* in additional PGC samples from control and GDM groups and confirmed a hypomethylation status at each detected CpG site and a marked decreasing in the mean methylation level of *Fyn* in GDM PGCs (Fig. 4a). To examine whether STZ would affect DNA methylation, PGCs of STZ-treated nondiabetic mice were collected, targeted bisulfite pyrosequencing of *Fyn* of this group showed no difference when comparing with control (Additional file 6: Fig. S6).

These results suggest that intrauterine hyperglycemia exposure leads to aberrant methylation erasure in D13.5 PGCs, and many of the differentially methylated genes were diabetes-related.

### Epigenetic alteration in F1 gametes was inherited by F2 somatic tissues

We performed RRBS in D13.5 PGCs, since global DNA methylation undergoes re-establishment at D18.5, methylation validation is necessary to investigate whether altered methylation erasure persists after global DNA methylation re-establishment process in later gametes. D18.5 testes from control and F1-GDM fetuses and sperm samples from the control and F1/C-GDM generation were used to investigate epigenetic maintenance in the F1 gametes. Then, D7.5 fetuses, adult liver, adipose tissue, and skeletal muscle from F2-GDM mice were used to investigate the effects of epigenetic inheritance from F1 to F2 generation. Finally, D13.5 PGCs, D18.5 testes from F2-GDM mice, sperm from F2/C-GDM mice, and D7.5 fetuses from F3-GDM were used to investigate the effects of epigenetic inheritance from F2 to F3 generation.

In these experiments, *Fyn* was investigated as a target gene. In F1 generation, D18.5 testes from F1-GDM fetuses exhibited decreased overall methylation as well as reduced methylation levels at CpG sites 1, 2, and 3 of *Fyn* (Fig. 4b). In sperm samples, the F1/C-GDM group had significantly lower methylation levels at all five CpG sites of *Fyn* (Fig. 4c). The mean methylation level at these five sites of *Fyn* was also lower than control group (Fig. 4c). Consistently decreased methylation levels of *Fyn* in D13.5 PGCs, D18.5 testes, and adult sperm from F1/C-GDM mice indicate the strong impact of intrauterine hyperglycemia exposure on DNA methylation status and suggest that alterations in early germ cells can be maintained in mature gametes in adulthood.

**Fig. 5:**
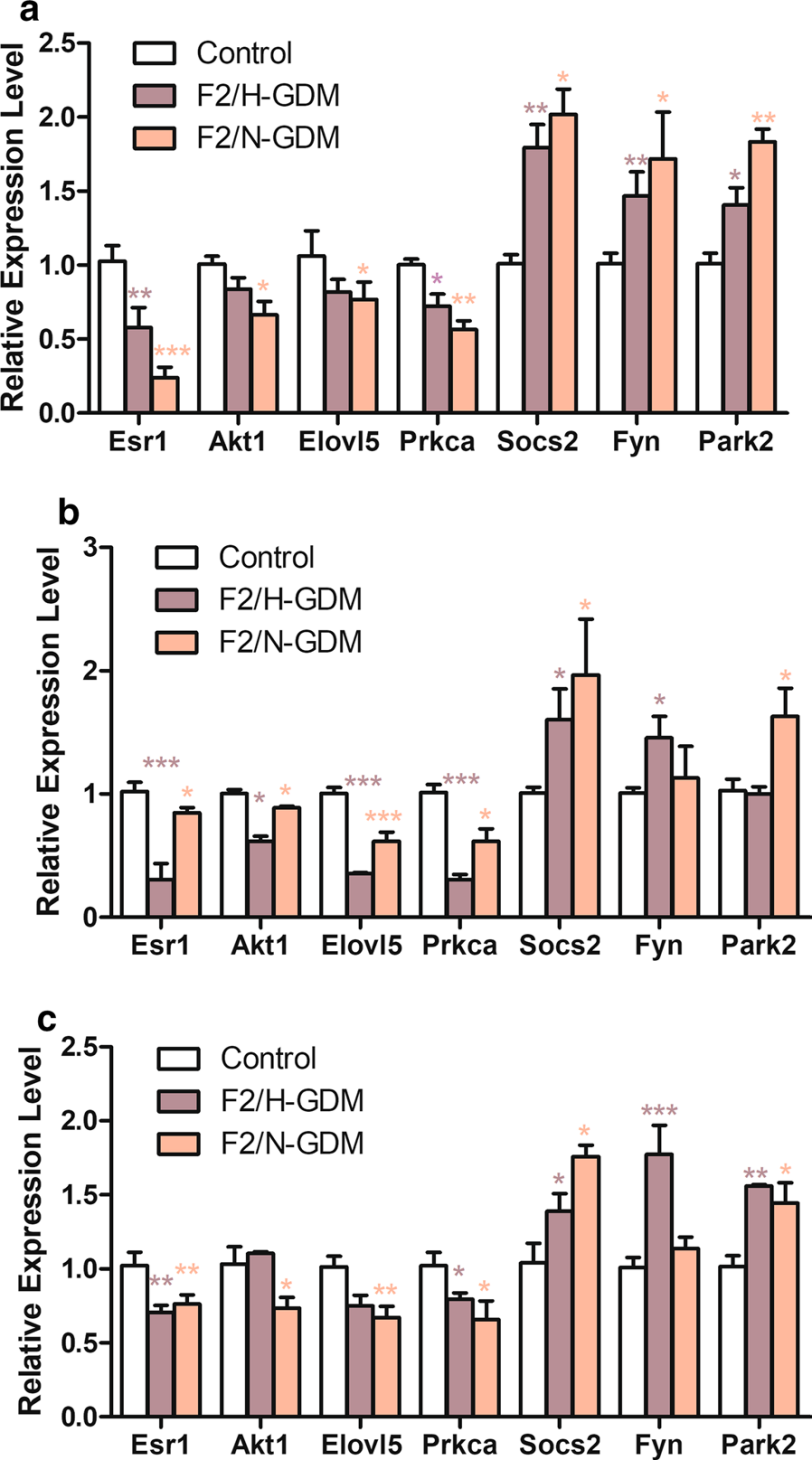
Relative mRNA expression of targeted DMGs in control and F2-GDM mice. Gene expression levels in adipose (**a**), liver (**b**), and muscle (**c**) tissues from control, F2/H-GDM, and F2/N-GDM male mice at 8 weeks (*n* = 5 mice per group). Data are presented as mean ± SEM. *p < 0.05, **p < 0.01, ***p < 0.001 versus the corresponding control

In F2 generation, D7.5 F2-GDM fetuses exhibited a significantly lower methylation level of *Fyn* compared with the control fetus (Fig. 4d). Based on the different phenotypes in F2 adult individuals, the F2/H-GDM and F2/N-GDM groups were analyzed separately. Liver, adipose, and skeletal muscle tissues were used since they are the main insulin target tissues. In visceral adipose tissues, F2/H-GDM and F2/N-GDM groups both exhibited significantly lower methylation at single sites and reduced overall methylation of *Fyn* compared with the control group (Fig. 4e). In addition, qPCR analysis of *Fyn* in adipose tissues from the F2/H-GDM and F2/N-GDM groups revealed significantly increased expression compared with control (Fig. 5a).

In the liver, the F2/H-GDM group exhibited significant hypomethylation at all five CpG sties (Fig. 4f), and *a* > 20% reduced overall methylation rate of *Fyn* compared with control (Fig. 4f). In addition, the qPCR for *Fyn* revealed significantly increased expression in the F2/H-GDM group compared with control (Fig. 5b). However, the F2/N-GDM group showed no DNA methylation or mRNA expression significance of *Fyn* in liver tissues compared with control (Figs. 4f, 5b).

In muscle, the F2/H-GDM group exhibited lower overall methylation levels as well as at all five CpG sites of *Fyn* compared with control (Fig. 4g), and mRNA expression of *Fyn* was increased compared with the control group (Fig. 5c). In contrast, there were no differences in methylation or expression of *Fyn* between the F2/N-GDM and control groups (Figs. 4g, 5c). Methylation at these loci is likely to inhibit transcriptional activity, since low methylation levels are associated with high expression levels.

We finally measured *Fyn* methylation levels in D13.5 PGCs and D18.5 testes of F2-GDM, but found no difference between the F2-GDM and control groups (Fig. 4b). Sperm from F2/C-GDM mice consistently exhibited no methylation difference in *Fyn* when comparing with the control (Fig. 4c). Further, D7.5 F3-GDM fetuses also exhibited no methylation difference of *Fyn* when comparing with the control (Fig. 4d). Methylation assessment on germ cells of F2-GDM and fetus of F3-GDM provides epigenetic evidence that intrauterine hyperglycemia exposure might have no effect on F2 germ cells or F3 generation.

We also performed qPCR analysis of the other six candidate genes in the F2 generation to indirectly assess the relationship between the epigenetic reprogramming and corresponding gene expression. Four hypermethylated genes (*Esr1*, *Akt1*, *Elovl5*, and *Prkca*) and three hypomethylated genes (*Socs2*, *Fyn*, and *Park2*) from RRBS array were validated. The mRNA relative expression levels of these seven genes were examined in adipose (Fig. 5a), liver (Fig. 5b), and muscle (Fig. 5c) from male F2 generation mice. Compared with control, hypermethylated genes *Esr1* and *Prkca* showed decreased mRNA expression in adipose, liver, and muscle of both F2/H- and F2/N-GDM groups; hypermethylated gene *Akt1* and *Elovl5* showed decreased expression in all three tissues of F2/N-GDM mice and significantly decreased expression in liver of F2/H-GDM mice; hypomethylated gene Socs2 showed increased expression in adipose, liver, and muscle of both F2/H- and F2/N-GDM groups; hypomethylated gene *Fyn* showed increased expression in all three tissues of F2/H-GDM mice and significantly increased expression only in adipose of F2/N-GDM mice; hypomethylated gene *Park2* showed increased expression in all three tissues of F2/N-GDM mice, and significantly increased expression only in adipose and muscle of F2/H-GDM mice. In general, the hypomethylated genes exhibited increased expression levels whereas the hypermethylated genes exhibited decreased expression (Fig. 5).

Together, these results suggest that the aberrant methylation caused by intrauterine hyperglycemia exposure in PGCs can be passed to fetal testes and mature sperm. In addition, this is a strong factor in determining the methylation status in somatic tissues but not germ cells of the F2 generation.

### Intrauterine hyperglycemia exposure had no effect on the F3 generation

**Fig. 6:**
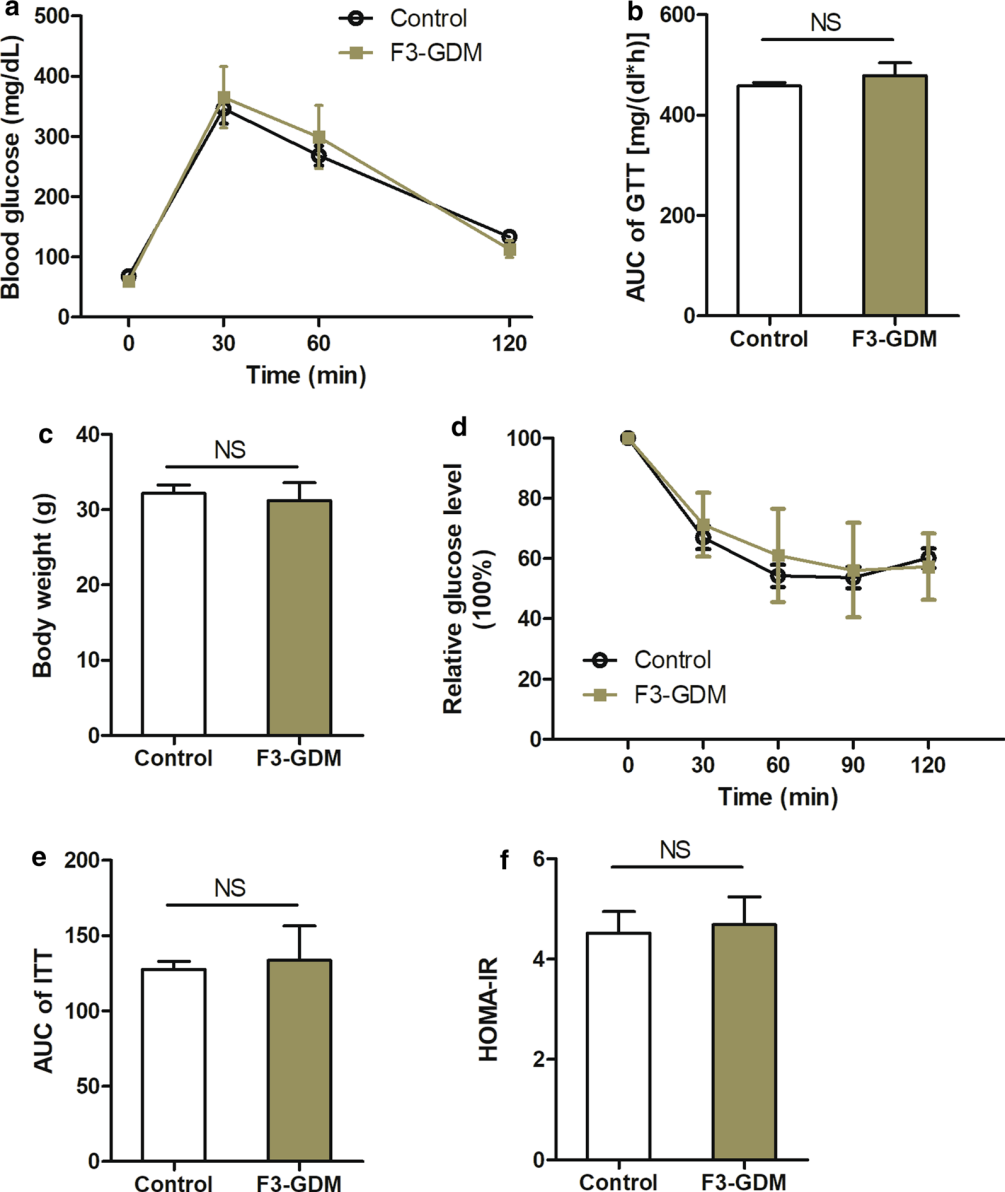
F3-GDM mice demonstrated no metabolic change at adulthood. **a** GTT curve and **b** the resulting AUC of control and F3-GDM male mice at 8 weeks. **c** Bodyweight of control and F3-GDM male mice at 8 weeks. **d** ITT curve and **e** the resulting AUC of control and F3-GDM male mice at 8 weeks. **f** HOMA-IR index of control and F3-GDM male mice at 8 weeks. For each group in **a**–**f**, control (*n* = 10), F3-GDM (*n* = 10). Data are presented as mean ± SEM

Based on the methylation assessment on F2-GDM germ cells and F3-GDM fetuses described above, the effects of intrauterine hyperglycemia exposure on the F3-GDM phenotypes were examined. F2-GDM mice without any abnormal phenotype were selected to produce F3-GDM progeny. At 8 weeks of age, there were no differences in GTT, ITT, bodyweight, HOMA-IR index, or serum biochemical parameters between F3-GDM and control mice (Fig. 6, Table 1). These results suggest that although intrauterine hyperglycemia exposure per se has a strong effect on the F2 generation, it has no effect on the F3 generation. This clarifies the role of intrauterine hyperglycemia exposure in intergenerational inheritance.

## Discussion

The present study demonstrated that intrauterine hyperglycemia affected the metabolic condition of the F2 generation but not the F3 generation and that the phenotype alteration of the F2 generation could be related to aberrant methylation erasure on day 13.5 PGCs of the F1 generation. These results provide clear evidence for a gamete-based origin of adult disease and highlight the importance of distinguishing the intergenerational effect caused by in utero exposure and postnatal exposure. Schematic diagram of this study was presented as Additional file 7: Fig. S7.

There were no significant differences in metabolic parameters between the F1/C-GDM male mice that were used as founders in the present study and control mice. Thus, we excluded adult influence factors, such as obesity, glucose metabolism dysfunction, and lipid metabolism dysfunction, which can be inheritable to progeny [19]. F2 male offspring of F1/C-GDM fathers exhibited impaired insulin sensitivity and increased body weight compared with control mice, and some F2 offspring developed impaired glucose tolerance at 8 weeks of age. These dysfunctions in the F2 offspring clearly demonstrated that intrauterine hyperglycemia exposure had a strong impact on grandchildren directly.

A previous study showed that the F2 offspring of F1-GDM fathers with IGT developed impaired glucose tolerance and decreased fasting insulin levels at 8 weeks, without alterations in bodyweight and fasting glucose levels [14]. The different metabolic features of the F2 generation and the F1 father indicate that both intrauterine exposure and postnatal metabolic exposure play a role in determining the acquired features of the progeny.

In the F1 generation, although F1-GDM mice exhibited glucose intolerance, none showed insulin resistance or increased bodyweight in the current study or previous reports [14]. Surprisingly, the F2 generation of healthy fathers had more diabetic phenotypes than the IGT F1-GDM mice in the current study. Thus, we speculate that intrauterine hyperglycemia may affect somatic cells and germ cells of the first generation differently, which could be caused by unsynchronized methylation reprogramming in somatic and germ cells [21]. In addition, this suggests that intrauterine exposure may have an impact on grandchildren well beyond direct effects on the F1 generation.

The current study revealed no metabolic changes in the F3-GDM generation born to healthy F2-GDM fathers. There was also no difference in the methylation status of *Fyn* in germ cells from F2/C-GDM mice or fetuses from the F3-GDM mice. These results suggest that aberrant methylation in gametes caused by intrauterine exposure can affect the F2 generation but is not heritable to the F3 generation.

Insulin resistance is a condition in which elevated insulin levels are required to maintain normal blood glucose. It is generally present in individuals with obesity and type II diabetes. Both genetic and environmental factors play a role in the development of insulin resistance [36–40]. Lipid accumulation is often coupled to an insulin resistant state [41]. When we compared the bodyweight of F1 and F2 generations of GDM, we supposed that abnormal lipid accumulation may be an important indicator of a severe metabolic dysfunction outcome in the F2 generation. Taking the phenotype traits and IPA analysis together, we used *Fyn* as a candidate gene to study methylation and intergenerational inheritance in this study. *Fyn* is involved in the control of metabolism, inflammation, adipogenesis, and insulin signaling. Available data indicate a strong link between *Fyn* and the development of diabetes [34, 42]. In the current study, we observed consistent hypomethylation of *Fyn* from fetal PGCs to mature sperm in F1/C-GDM mice, then inherited by F2 somatic cells but not germ cells, and finally exhibited no methylation alteration in F3 generation. The methylation status of *Fyn* was synchronous with metabolic phenotypes presented in offspring, therefore providing a link between metabolic changes and epigenetic alteration in intergenerational transmission.

Overall, this study uniquely characterized intrauterine hyperglycemia exposure as an important determinant of diabetic outcome in F2 male offspring independent of postnatal environmental exposure, but not a weighting factor on metabolic and epigenetic changes in the F3 generation. In addition, intrauterine hyperglycemia exposure could induce aberrant methylation in PGCs as early as D13.5 during pregnancy.

It may be useful to track dynamic methylation status of from single embryo to adulthood individually. Unfortunately, up to date, there is no suitable way to do it. We hope that it could be performed in future.

## Conclusion

Our data suggest that intrauterine hyperglycemia exposure alters DNA methylation patterns in D13.5 primordial germ cells of F1 generation, which could be maintained in somatic cells but not germ cells in F2 generation, thus causing an intergenerational inheritance of metabolic disorder in F2 but not F3 generation. Our results indicate the necessity of subdividing the exposure history occurred at different periods in life span and reconsider the origin of epigenetic and metabolic inheritance on F3 generation when studying DOHaD.

## Additional files

**Additional file 1.** Primers designed for pyrosequencing and qPCR in the study, and gender confirmation of D13.5 male PGCs.

**Additional file 2.** Metabolic phenotypes and bio-parameters of F2/C-GDM male mice at 8 weeks.

**Additional file 3.** Distribution of differentially methylated loci in gene elements, and hierarchical clustering presentation of RRBS sequencing.

**Additional file 4.** Differentially methylated genes in D13.5 PGCs of F1-GDM mice.

**Additional file 5.** IPA pathway analysis of RRBS methylome sequencing.

**Additional file 6.** DNA methylation validation in control and STZ-treated nondiabetic mice.

**Additional file 7.** Schematic diagram of the study

## Abbreviations

DOHaD: developmental origins of health and disease
GDM: gestational diabetes mellitus
PGCs: primordial germ cells
STZ: streptozotocin
GTT: glucose tolerance test
ITT: insulin tolerance test
AUC: area under the curve
TG: triglyceride
TC: total cholesterol
HDL: high-density lipoprotein
LDL: low-density lipoprotein
HOMA-IR: homeostasis model of insulin resistance
RRBS: reduced representation bisulfite sequencing
DML: differentially methylated loci
DMRs: differentially methylated regions
IPA: Ingenuity Pathway Analysis
