# Supplementary material for: Intrauterine hyperglycemia exposure results in intergenerational inheritance via DNA methylation reprogramming on F1 PGCs
Source: Epigenetics Chromatin. 2018 May 25;11:20. doi: 10.1186/s13072-018-0192-2 (PMC5968593; doi:10.1186/s13072-018-0192-2)
Supplement: Supplementary file 1 — Additional file 1. Primers designed for pyrosequencing and qPCR in the study, and gender confirmation of D13.5 male PGCs. [file 13072_2018_192_MOESM1_ESM.pdf]

Additional file 1
Primers for pyrosequencing PCR

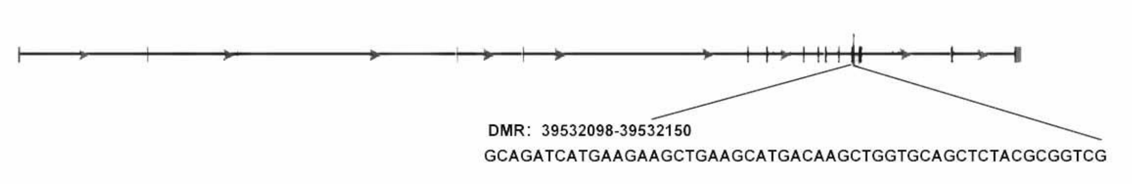

Figure S1. Gene model of *Fyn*.

The region examined by pyrosequencing is at Chr10: 39531998-39532250. Five CpG sites were included in the Fyn pyrosequencing. The primers for Fyn bisul fite DNA PCR and sequencing were as follows: forward, 5′-TTTTTGGAGGAG GAGTAGATTATGAAGA-3′; reverse, 5′-Biot-AAAAAAAAAAATACCACTAACC TTTACTCA-3′; sequencing primer, 5′-ATGATAAGTTGGTGTAGT-3′.

Primers for qPCR analysis

The following primers were used for qPCR amplication: *Sry* forward, 5′-ATGT CAAGCGCCCCATGAAT-3′; *Sry* reverse, 5′-CCCTCCGATGAGGCTGATATTTA-3′; *Esr1* forward, 5′-CATGGTCATGGTAAGTGGCA-3′; *Esr1* reverse, 5′-TCTCT GGGCGACATTCTTCT-3′; *Akt1* forward, 5′-CCATCGTTCTTGAGGAGGAA-3′; *Akt1* reverse, 5′-CATGAACGACGTAGCCATTG-3′; *Elovl5* forward, 5′-CTGAGT GACGCATCGAAATG-3′; *Elovl5* reverse, 5′-CTTGCACATCCTCCTGCTC-3′; *So cs2* forward, 5′-AAGAAAGTTCCTTCTGGAGCC-3′; *Socs2* reverse, 5′-CGCGA GCTCAGTCAAACAG-3′; *Fyn* forward, 5′-ATCCAAGCTCCCAAATTTCC-3′; *F yn* reverse 5′-TGCTGATCTAATCGTGGCAA-3′; *Park2* forward, 5′-ATCGACCT CCACTGGGAAG-3′; *Park2* reverse, 5′-GCGTAGGTCCTTCTCGACC-3′; *Prkca* forward 5′-AACGAACTCATGGCACCTCT-3′; *Prkca* reverse, 5′-CACTGCACCG ACTTCATCTG-3′; *β-actin* forward, 5′-GGCTGTATTCCCCTCCATCG-3′; *β-actin* reverse, 5′-CCAGTTGGTAACAATGCCATGT-3′.

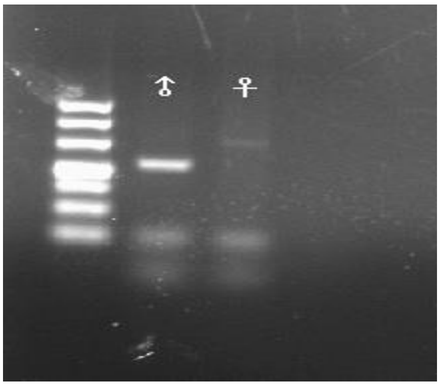

Figure S3. Gender confirmation of D13.5 PGCs by Sry.
