# Supplementary material for: Intrauterine hyperglycemia exposure results in intergenerational inheritance via DNA methylation reprogramming on F1 PGCs
Source: Epigenetics Chromatin. 2018 May 25;11:20. doi: 10.1186/s13072-018-0192-2 (PMC5968593; doi:10.1186/s13072-018-0192-2)
Supplement: Supplementary file 2 — Additional file 2. Metabolic phenotypes and bio-parameters of F2/C-GDM male mice at 8 weeks. [file 13072_2018_192_MOESM2_ESM.pdf]

Additional file 2

Table S1. Serum biochemical parameters in control and F2/C-GDM male mice at 8 weeks.

	**Control (n=10)**	**F2/C-GDM (n=6)**
Fasting Glucose (mM)	4.57±0.23	4.37±0.26
Fasting insulin (mIU/l)	22.05±6.97	22.49±4.30
TC (mM)	2.47±0.06	2.27±0.18
TG (mM)	1.33±0.11	1.69±0.16
HDL (mM)	1.39±0.03	1.58±0.11
LDL (mM)	0.20±0.01	0.24±0.03

TC, total cholesterol; TG, triacylglycerol; HDL, high density lipoprotein; LDL, low density lipoprotein. All parameters were measured at 8 weeks of age. Values are expressed as mean ± S.E.M.

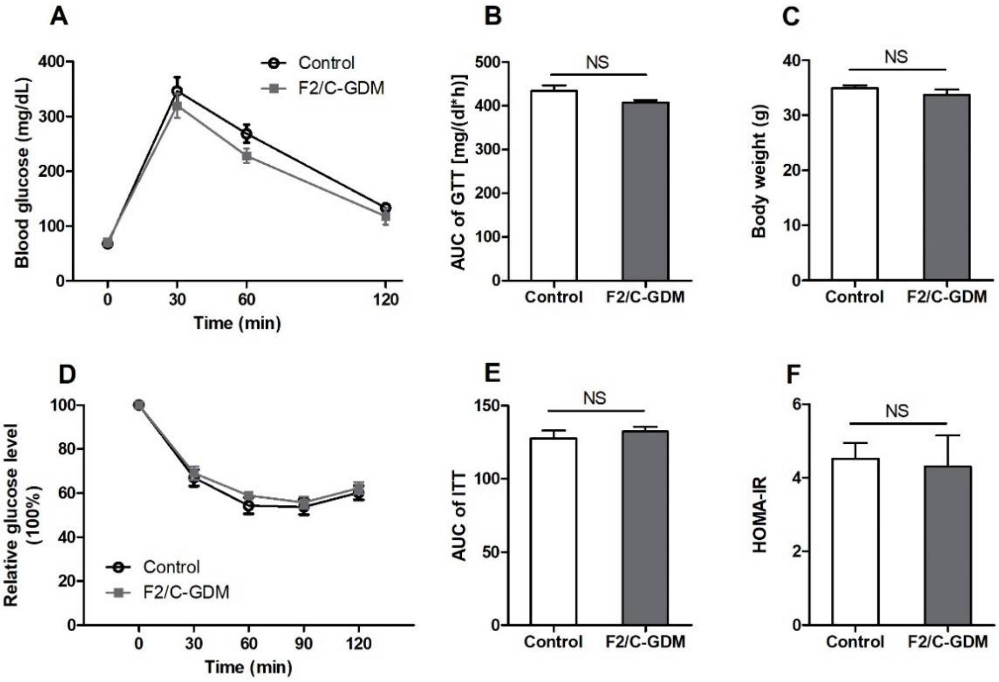

Figure S2. F2/C-GDM mice demonstrated an unaffected metabolic phenotype at adulthood. (**A**) GTT curve and (**B**) the resulting AUC of control (n=10) and F2/C-GDM (n=6) male mice at 8 weeks. (**C**) Bodyweight of control (n=10) and F2/C-GDM (n=6) male mice at 8 weeks. (**D**) ITT curve and (**E**) the resulting AUC of control (n=10) and F2/C-GDM (n=6) male mice at 8 weeks. (**F**) HOMA-IR index of control (n=10) and F2/C-GDM (n=6) male mice at 8 weeks. Data are presented as means ± S.E.M.
