# Supplementary material for: Intrauterine hyperglycemia exposure results in intergenerational inheritance via DNA methylation reprogramming on F1 PGCs
Source: Epigenetics Chromatin. 2018 May 25;11:20. doi: 10.1186/s13072-018-0192-2 (PMC5968593; doi:10.1186/s13072-018-0192-2)
Supplement: Supplementary file 3 — Additional file 3. Distribution of differentially methylated loci in gene elements, and hierarchical clustering presentation of RRBS sequencing. [file 13072_2018_192_MOESM3_ESM.pdf]

Additional file 3

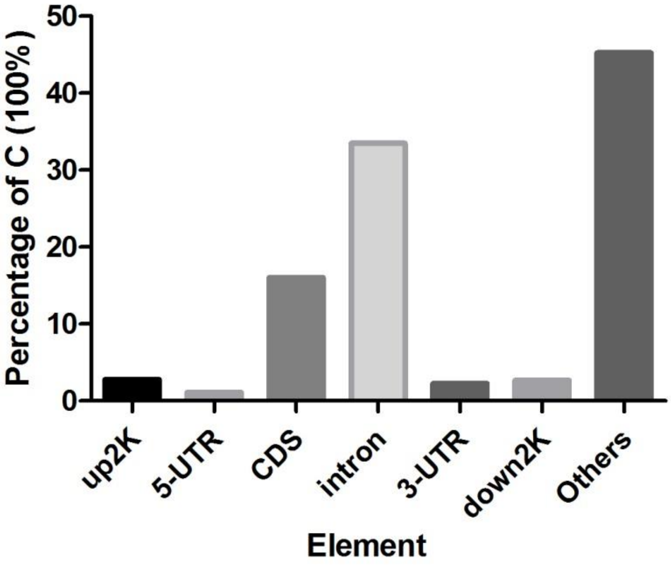

Figure S4. Distribution of differentially methylated loci in gene elements.

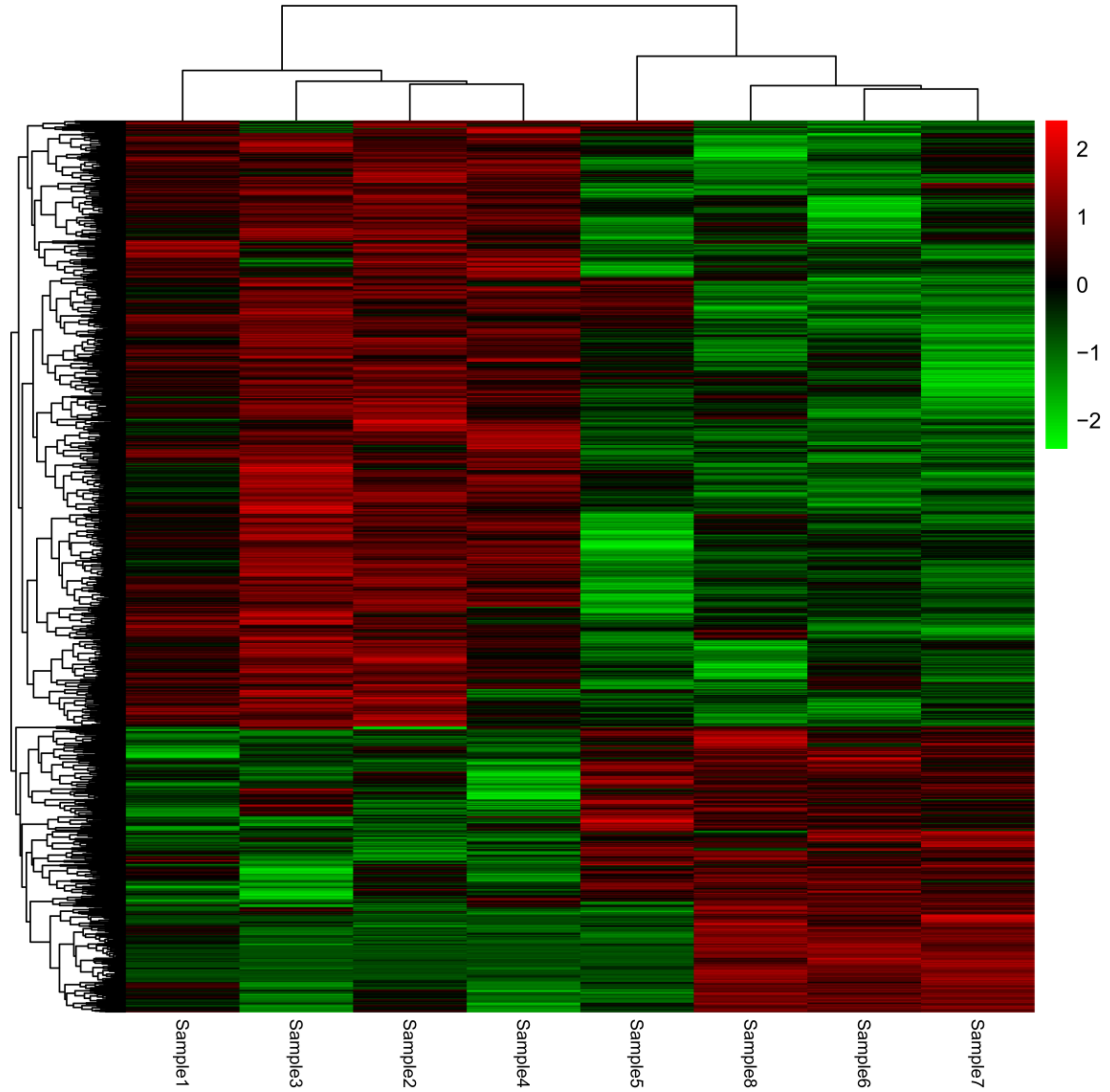

Figure S5. Hierarchical clustering presentation of RRBS sequencing in PGCs. Each horizonal row represents one differentially methylated loci, each column represents one sample. Sample1-4: control group, sample 5-8: GDM group. Increasing green intensities denote loci that was hypomethylated, and increasing red intensities denote loci that was hypermethylated in GDM samples compared with control samples.
