# Supplementary material for: Intrauterine hyperglycemia exposure results in intergenerational inheritance via DNA methylation reprogramming on F1 PGCs
Source: Epigenetics Chromatin. 2018 May 25;11:20. doi: 10.1186/s13072-018-0192-2 (PMC5968593; doi:10.1186/s13072-018-0192-2)
Supplement: Supplementary file 4 — Additional file 4. Differentially methylated genes in D13.5 PGCs of F1-GDM mice. [file 13072_2018_192_MOESM4_ESM.pdf]

Additonal file 4
Table S2. DMRs related gene of RRBS
meanMethy1:control meanMethy2:GDM

DMR location										DMR location in gene elements					
#chr	start	end	length	Num_C	meanMethy1	meanMethy2	diff_Methy	Symbol	Up2K	5-UTR	CDS	Intron	3-UTR	Down2K
chr12	112663413	112663466	54	8	0.43166304	0.65468761	-0.22302	Akt1	NO	NO	NO	YES	NO	NO
chr17	11500291	11500376	86	4	0.70166625	0.03542468	0.666242	Park2	NO	NO	NO	YES	NO	NO
chr10	39532098	39532150	53	4	0.79191796	0.30847997	0.483438	Fyn	NO	NO	YES	NO	NO	NO
chr9	77953649	77953734	86	4	0.30219218	0.60395812	-0.30177	Elovl5	NO	NO	NO	YES	NO	NO
chr10	95414942	95415007	66	5	0.38773599	0.00395372	0.383782	Socs2	NO	YES	NO	NO	NO	NO
chr11	107975315	107975386	72	6	0.17004382	0.72288422	-0.55284	Prkca	NO	NO	NO	YES	NO	NO
chr10	4870065	4870214	150	4	0.19798018	0.69591668	-0.49794	Esr1	NO	NO	NO	YES	NO	NO
chr4	116323464	116323548	85	4	0.20082668	0.81411893	-0.61329	Mast2	NO	NO	NO	YES	NO	NO
chr5	135745072	135745173	102	4	0.46671388	0.26028062	0.206433	Tmem120a	YES	NO	NO	NO	NO	NO
chr6	97594820	97594920	101	4	0.25687627	0.54378333	-0.28691	Frmd4b	NO	NO	NO	YES	NO	NO
chr5	144845326	144845394	69	6	0.37079917	0.74721021	-0.37641	Trrap	NO	NO	NO	YES	NO	NO
chr12	69594970	69595100	131	9	0.56278909	0.77797363	-0.21518	Sos2	NO	NO	NO	YES	NO	NO
chr5	137295953	137296013	61	5	0.38110494	0.70005757	-0.31895	Ufsp1	NO	NO	NO	NO	NO	YES
chr10	80435359	80435468	110	5	0.59923479	0.69855174	-0.09932	Tcf3	YES	NO	NO	NO	NO	NO
chr9	56886963	56887112	150	14	0.47360426	0.74717349	-0.27357	Cspg4	NO	NO	YES	NO	NO	NO
chr10	43511229	43511338	110	9	0.78266123	0.68576098	0.0969	Bend3	NO	NO	YES	NO	NO	NO
chr1	15497499	15497623	125	18	0.7991696	0.48922316	0.309946	Kcnb2	NO	NO	NO	YES	NO	NO
chr8	58471428	58471478	51	7	0.77685689	0.69714635	0.079711	Galntl6	NO	NO	NO	YES	NO	NO
chr6	145136033	145136095	63	5	0.13067423	0.2380468	-0.10737	Lrmp	NO	NO	NO	YES	NO	NO
chr3	37225278	37225371	94	9	0.62491323	0.60813175	0.016781	Il21	NO	NO	YES	NO	YES	NO
chr17	27982137	27982200	64	4	0.60826619	0.46778934	0.140477	Anks1	NO	NO	NO	YES	NO	NO
chr11	120010112	120010222	111	7	0.62196495	0.60489589	0.017069	Aatk	NO	NO	YES	NO	NO	NO
chr1	25591063	25591148	86	4	0.70035425	0.48836487	0.211989	Adgrb3	NO	NO	NO	YES	NO	NO
chr16	30959937	30959995	59	8	0.44002275	0.73276965	-0.29275	Mir1195	NO	NO	NO	YES	NO	NO
chr5	35908905	35908996	92	4	0.1992697	0.25705027	-0.05778	Afap1	NO	NO	NO	YES	NO	NO
chr18	56736249	56736303	55	5	0.84303513	0.74165329	0.101382	Lmnb1	NO	NO	NO	YES	NO	NO
chr2	165853815	165853965	151	7	0.17895411	0.64083544	-0.46188	Zmynd8	NO	NO	NO	YES	NO	NO
chr6	86756527	86756582	56	7	0.44517161	0.70574038	-0.26057	Anxa4	NO	NO	NO	YES	NO	NO
chr2	105143985	105144080	96	4	0.15677675	0.60576843	-0.44899	Wt1	NO	NO	NO	YES	NO	NO
chr5	143218979	143219046	68	7	0.44040604	0.7526793	-0.31227	Spdyb	NO	NO	YES	NO	NO	NO
chr3	83033822	83033913	92	4	0.87509789	0.66226136	0.212837	Fga	NO	NO	NO	NO	NO	YES
chr2	151706724	151706826	103	12	0.48159063	0.70237998	-0.22079	Tmem74b	NO	NO	YES	NO	NO	NO
chr11	50791879	50791970	92	8	0.44234375	0.34293883	0.099405	Adamts2	NO	NO	YES	NO	NO	NO

chr7	29382888	29382969	82	5	0.55154781	0.41593407	0.135614	Sipa1l3	NO	NO	NO	YES	NO	NO
chr17	86264491	86264575	85	4	0.7799337	0.65306965	0.126864	Prkce	NO	NO	NO	YES	NO	NO
chr9	89601504	89601575	72	7	0.54874957	0.74610559	-0.19736	AF529169	NO	NO	YES	NO	NO	NO
chr1	170329243	170329295	53	4	0.69500175	0.14100283	0.553999	Nos1ap	NO	NO	YES	NO	NO	NO
chr9	117118292	117118356	65	5	0.03791031	0.59176087	-0.55385	Rbms3	NO	NO	NO	YES	NO	NO
chr4	45920887	45920983	97	8	0.11612292	0.54106936	-0.42495	E230008N1	NO	NO	YES	NO	NO	NO
chr4	141845696	141845768	73	5	0.63907907	0.67376213	-0.03468	Ctrcos	NO	NO	NO	YES	NO	NO
chr11	97146544	97146615	72	5	0.90531591	0.38546111	0.519855	Mir7235	YES	NO	NO	NO	NO	NO
chr11	78516641	78516698	58	4	0.32132622	0.78763319	-0.46631	Poldip2	NO	NO	NO	YES	NO	NO
chr4	132230968	132231025	58	4	0.52305379	0.81063089	-0.28758	Gmeb1	NO	NO	YES	NO	NO	NO
chr17	46018803	46018919	117	7	0.48782086	0.6395951	-0.15177	Vegfa	NO	NO	YES	NO	NO	NO
chr8	36103920	36103973	54	4	0.03786844	0.54277978	-0.50491	D8Ertd82e	NO	NO	YES	NO	NO	NO
chr5	116228539	116228939	401	4	0.35830276	0.77603243	-0.41773	Ccdc60	NO	NO	NO	YES	NO	NO
chr9	114922959	114923043	85	6	0.68165143	0.74796768	-0.06632	Gpd1l	NO	NO	NO	YES	NO	NO
chr8	120040104	120040175	72	5	0.03786844	0.6088863	-0.57102	Crispld2	NO	NO	YES	NO	NO	NO
chr5	72957557	72957632	76	4	0.84112133	0.47567205	0.365449	Slain2	NO	NO	YES	NO	NO	NO
chr9	70903689	70903800	112	8	0.38308158	0.75757337	-0.37449	Lipc	NO	NO	NO	YES	NO	NO
chr11	98042766	98042833	68	6	0.28597692	0.59617567	-0.3102	Stac2	NO	NO	YES	NO	NO	NO
chr9	15315937	15315990	54	6	0.82811022	0.72692741	0.101183	Taf1d	NO	NO	NO	YES	NO	NO
chr17	56172131	56172182	52	5	0.83878708	0.6630199	0.175767	Tnfaip8l1	NO	NO	YES	NO	NO	NO
chr15	101924529	101924591	63	4	0.25931649	0.60336607	-0.34405	Krt4	NO	NO	YES	NO	NO	NO
chr6	96164781	96164867	87	6	0.15885734	0.4278757	-0.26902	1700123L1	NO	NO	NO	NO	YES	NO
chr5	65043846	65043949	104	4	0.29188385	0.63538493	-0.3435	Fam114a1	NO	NO	NO	NO	NO	YES
chr6	119524197	119524331	135	6	0.04321372	0.75638785	-0.71317	Wnt5b	NO	NO	NO	YES	NO	NO
chr7	139020834	139020964	131	9	0.72078837	0.17672955	0.544059	Jakmip3	NO	NO	YES	NO	NO	NO
chr15	81410512	81410565	54	5	0.8852887	0.77556204	0.109727	Xpnpep3	NO	NO	NO	YES	NO	NO
chr6	46153590	46153673	84	7	0.82429002	0.53398297	0.290307	Cntnap2	NO	NO	NO	YES	NO	NO
chr7	139125191	139125308	118	10	0.56906014	0.78344613	-0.21439	Stk32c	NO	YES	YES	NO	NO	NO
chr8	58388967	58389064	98	7	0.89167282	0.43661171	0.455061	Galntl6	NO	NO	NO	YES	NO	NO
chr11	23438649	23438712	64	4	0.20213031	0.77746552	-0.57534	Usp34	NO	NO	NO	YES	NO	NO
chr10	88423950	88424037	88	5	0.83144184	0.64296717	0.188475	Gnptab	NO	NO	NO	YES	NO	NO
chr4	151983269	151983320	52	5	0.37599907	0.48921579	-0.11322	Dnajc11	NO	NO	NO	NO	NO	YES
chr2	58468558	58468617	60	5	0.20405633	0.80596766	-0.60191	Acvr1	NO	NO	YES	NO	NO	NO
chr15	100936284	100936411	128	20	0.7401745	0.42637502	0.313799	Scn8a	NO	YES	YES	NO	NO	NO
chr14	63918769	63918830	62	11	0.42162499	0.73582106	-0.3142	Pinx1	NO	NO	NO	YES	NO	NO
chr8	122780547	122780630	84	5	0.53273337	0.71511923	-0.18239	Acsf3	NO	NO	YES	NO	NO	NO
chr10	96230483	96230578	96	6	0.90247329	0.63654194	0.265931	4930459C0	NO	NO	NO	YES	NO	NO
chr4	112955998	112956071	74	5	0.70159986	0.87237519	-0.17078	Skint6	NO	NO	NO	YES	NO	NO

chr18	77198544	77198595	52	7	0.1696435	0.44330428	-0.27366	St8sia5	NO	NO	NO	YES	NO	NO
chr5	147990871	147990962	92	5	0.85558945	0.4804247	0.375165	Mtus2	NO	NO	NO	YES	NO	NO
chr4	139578658	139578773	116	4	0.24287726	0.7189064	-0.47603	Iffo2	NO	NO	NO	YES	NO	NO
chr19	7096468	7096532	65	4	0.24577739	0.19288174	0.052896	Macrod1	NO	NO	NO	YES	NO	NO
chr12	31831346	31831423	78	11	0.43391249	0.72808056	-0.29417	Cog5	NO	NO	NO	YES	NO	NO
chr11	49625786	49625949	164	12	0.24634512	0.60530207	-0.35896	Flt4	NO	NO	NO	YES	NO	NO
chr5	137295953	137296013	61	5	0.38110494	0.70005757	-0.31895	Srrt	NO	NO	NO	YES	NO	NO
chr10	76294504	76294590	87	6	0.15524782	0.63183023	-0.47658	Dip2a	NO	NO	YES	NO	NO	NO
chr17	27719942	27720104	163	10	0.51876074	0.54519263	-0.02643	Spdef	NO	NO	YES	NO	NO	NO
chr4	139277111	139277201	91	5	0.7645659	0.54794457	0.216621	Capzb	NO	NO	NO	YES	NO	NO
chr8	108809275	108809325	51	4	0.20718916	0.63818088	-0.43099	Zfhx3	NO	NO	NO	YES	NO	NO
chrX	98741869	98741976	108	18	0.82036574	0.48950739	0.330858	Ophn1	NO	NO	NO	YES	NO	NO
chr5	115103427	115103508	82	8	0.89458625	0.61046194	0.284124	Rpl37rt	NO	NO	YES	NO	NO	NO
chr15	94547790	94547876	87	4	0.34257488	0.73660991	-0.39404	Irak4	NO	YES	NO	YES	NO	NO
chrX	164108338	164108448	111	6	0.32521859	0.77725889	-0.45204	Tmem27	NO	NO	YES	NO	NO	NO
chr12	112909195	112909269	75	13	0.5422949	0.7480701	-0.20578	Jag2	NO	NO	YES	NO	NO	NO
chr11	67083773	67083883	111	4	0.75325198	0.38011187	0.37314	Myh3	NO	NO	YES	NO	NO	NO
chr5	137059905	137059968	64	5	0.32045801	0.71134078	-0.39088	Serpine1	NO	NO	NO	NO	NO	YES
chr7	30523033	30523132	100	9	0.42627855	0.72822128	-0.30194	Arhgap33o	NO	NO	NO	NO	NO	YES
chr1	165668367	165668438	72	5	0.21153852	0.63433306	-0.42279	Rcsd1	NO	NO	NO	YES	NO	NO
chr7	81144674	81144782	109	9	0.20775384	0.46943258	-0.26168	Slc28a1	NO	NO	NO	YES	NO	NO
chr7	60005043	60005093	51	5	0.05695826	0.29948149	-0.24252	Snrpn	NO	YES	NO	YES	NO	NO
chr5	142042391	142042491	101	9	0.10136378	0.31081902	-0.20946	Sdk1	NO	NO	NO	YES	NO	NO
chr2	179980094	179980175	82	4	0.03400265	0.46243432	-0.42843	4921531C2	NO	NO	NO	NO	NO	YES
chr5	131717819	131717895	77	7	0.59789061	0.45860864	0.139282	Auts2	NO	NO	NO	YES	NO	NO
chr7	28559646	28559748	103	15	0.27931888	0.63630175	-0.35698	Pak4	NO	NO	YES	NO	NO	NO
chr4	56967901	56968022	122	4	0.24310226	0.74602758	-0.50293	Frrs1l	NO	NO	NO	YES	NO	NO
chr3	152398833	152398896	64	4	0.25689021	0.76820668	-0.51132	Zzz3	NO	NO	NO	YES	NO	NO
chr7	100497220	100497316	97	13	0.64335983	0.73446436	-0.0911	Ucp2	NO	NO	YES	NO	NO	NO
chr8	70780345	70780452	108	4	0.07799978	0.5421634	-0.46416	Mast3	NO	NO	YES	NO	NO	NO
chr3	135520213	135520283	71	7	0.85292602	0.68352701	0.169399	Manba	NO	NO	NO	YES	NO	NO
chr17	34978300	34978458	159	21	0.19928374	0.56895216	-0.36967	Hspa1l	NO	NO	YES	NO	NO	NO
chr16	10449070	10449145	76	5	0.12808247	0.71515849	-0.58708	Tvp23a	YES	NO	NO	NO	NO	NO
chr19	53018715	53018779	65	5	0.88552652	0.56580766	0.319719	Xpnpep1	NO	NO	NO	YES	NO	NO
chr1	181835233	181835285	53	8	0.25064749	0.57137105	-0.32072	Lbr	NO	NO	NO	YES	NO	NO
chr9	114724890	114724940	51	5	0.61111669	0.46079486	0.150322	Dync1li1	NO	NO	NO	NO	NO	YES
chr11	3150670	3150794	125	13	0.50800474	0.79379078	-0.28579	Sfi1	NO	NO	NO	YES	NO	NO
chr2	32208671	32208801	131	13	0.41115637	0.77288101	-0.36172	Prrc2b	NO	NO	YES	NO	NO	NO

chr6	99009762	99009881	120	6	0.05996717	0.36522505	-0.30526	Foxp1	NO	NO	NO	YES	NO	NO
chr6	107568296	107568411	116	4	0.04321372	0.77253258	-0.72932	Lrrn1	NO	NO	YES	NO	NO	NO
chr5	136219097	136219236	140	7	0.1835658	0.60665321	-0.42309	Sh2b2	NO	NO	NO	YES	NO	NO
chr6	72679027	72679083	57	4	0.69510342	0.41061205	0.284491	Tcf7l1	NO	NO	NO	YES	NO	NO
chr4	134243869	134243965	97	5	0.33779113	0.75431659	-0.41653	Zfp593	NO	NO	NO	NO	YES	NO
chr18	13911749	13911820	72	4	0.10438158	0.43510514	-0.33072	Zfp521	NO	NO	NO	YES	NO	NO
chr5	135349852	135349997	146	33	0.33867879	0.62591943	-0.28724	Fkbp6	YES	NO	YES	YES	NO	NO
chr11	6007122	6007236	115	14	0.29848608	0.0099185	0.288568	Camk2b	NO	NO	NO	YES	NO	NO
chr5	114578331	114578411	81	5	0.37981495	0.34227235	0.037543	Fam222a	NO	NO	NO	YES	NO	NO
chr4	148008947	148009016	70	10	0.45431078	0.71752045	-0.26321	Clcn6	NO	NO	YES	NO	NO	NO
chr2	129125879	129125958	80	5	0.24759547	0.75845827	-0.51086	Polr1b	NO	NO	YES	NO	NO	NO
chr18	78858998	78859052	55	5	0.5500515	0.80460469	-0.25455	Setbp1	NO	NO	YES	NO	NO	NO
chr10	91001665	91001772	108	7	0.32722333	0.29540649	0.031817	Apaf1	NO	NO	NO	YES	NO	NO
chr1	180162212	180162338	127	8	0.90867114	0.66415681	0.244514	Cdc42bpa	NO	NO	NO	NO	YES	NO
chr11	116077411	116077533	123	6	0.60149693	0.46794545	0.133551	Unc13d	NO	NO	YES	NO	NO	NO
chr5	137295953	137296013	61	5	0.38110494	0.70005757	-0.31895	Mir7036	NO	NO	NO	NO	NO	YES
chr2	49741796	49741899	104	5	0.8554212	0.58462204	0.270799	Kif5c	NO	NO	NO	YES	NO	NO
chr13	51755074	51755161	88	4	0.23104343	0.61259252	-0.38155	Sema4d	NO	NO	NO	YES	NO	NO
chr19	41747119	41747516	398	6	0.84209578	0.58335573	0.25874	Gm19424	NO	NO	NO	YES	NO	NO
chr5	116143059	116143875	817	8	0.63471574	0.73558482	-0.10087	Ccdc60	NO	NO	NO	YES	NO	NO
chr7	30523033	30523132	100	9	0.42627855	0.72822128	-0.30194	Arhgap33	NO	NO	YES	NO	NO	NO
chr11	22973087	22973161	75	11	0.19354835	0.35044017	-0.15689	Commd1	NO	NO	NO	YES	NO	NO
chr5	103261469	103261550	82	10	0.46460124	0.71438107	-0.24978	4930429D1	NO	NO	NO	YES	NO	NO
chr17	85286743	85286885	143	5	0.2060919	0.69668832	-0.4906	Camkmt	NO	NO	NO	YES	NO	NO
chr8	108775112	108775173	62	5	0.74383254	0.50719968	0.236633	Zfhx3	NO	NO	NO	YES	NO	NO
chr10	121435437	121435574	138	5	0.12841469	0.45329844	-0.32488	Rassf3	NO	NO	NO	YES	NO	NO
chr2	31044865	31044926	62	6	0.17499122	0.51211735	-0.33713	Fnbp1	NO	NO	YES	YES	NO	NO
chr4	128395005	128395166	162	24	0.43631633	0.67858366	-0.24227	Csmd2	NO	NO	YES	NO	NO	NO
chr19	38140189	38140256	68	5	0.5576645	0.72156952	-0.16391	Pde6c	NO	NO	YES	YES	NO	NO
chr9	114096418	114096853	436	4	0.56165674	0.60636691	-0.04471	Susd5	NO	NO	YES	NO	NO	NO
chr12	75362656	75362721	66	7	0.62198984	0.58688627	0.035104	Rhoj	NO	NO	NO	YES	NO	NO
chr8	77573730	77573850	121	5	0.32426327	0.70919731	-0.38493	Prmt10	NO	NO	NO	YES	NO	NO
chr10	122826116	122826246	131	6	0.87756331	0.66910597	0.208457	Ppm1h	NO	NO	NO	YES	NO	NO
chr16	75765001	75765124	124	5	0.1989039	0.71903822	-0.52013	Hspa13	NO	NO	YES	NO	YES	NO
chr6	5821971	5824545	2575	17	0.75711673	0.70295595	0.054161	Dync1i1	NO	NO	NO	YES	NO	NO
chr5	129901967	129902018	52	4	0.78631471	0.65184657	0.134468	Zbed5	NO	NO	YES	NO	NO	NO
chr8	14846638	14846730	93	13	0.45775341	0.68395628	-0.2262	Dlgap2	NO	NO	YES	NO	NO	NO
chr13	42156325	42156388	64	5	0.32703681	0.68957901	-0.36254	Hivep1	NO	NO	YES	NO	NO	NO

chr4	112393269	112393375	107	10	0.32720913	0.64328639	-0.31608	Skint9	NO	NO	NO	YES	NO	NO
chr5	142422324	142422383	60	6	0.88715597	0.68686503	0.200291	Foxk1	NO	NO	NO	YES	NO	NO
chr4	85035445	85035525	81	11	0.66712945	0.82485065	-0.15772	Cntln	NO	NO	NO	YES	NO	NO
chr11	102357217	102357301	85	7	0.55785856	0.39018548	0.167673	Slc4a1	NO	NO	NO	YES	NO	NO
chr1	75405015	75405107	93	7	0.41585104	0.81867931	-0.40283	Speg	NO	NO	YES	NO	NO	YES
chr10	62562727	62562903	177	6	0.37835339	0.75594753	-0.37759	2510003E0	NO	NO	NO	YES	NO	NO
chr5	134670485	134670576	92	11	0.84780422	0.71084215	0.136962	Limk1	NO	NO	NO	YES	NO	NO
chr7	68317656	68317777	122	4	0.03814266	0.65060513	-0.61246	Fam169b	NO	NO	NO	YES	NO	NO
chr1	171418160	171418309	150	6	0.32318658	0.57858059	-0.25539	Usf1	NO	NO	NO	NO	YES	NO
chr2	165823475	165823551	77	6	0.64352549	0.74660226	-0.10308	Zmynd8	NO	NO	NO	YES	NO	NO
chr4	60619011	60619106	96	7	0.498437	0.80172367	-0.30329	Mup2	NO	NO	NO	YES	NO	NO
chr12	73288437	73288487	51	5	0.46339863	0.75347168	-0.29007	Trmt5	YES	NO	NO	NO	NO	NO
chr8	11732290	11732342	53	4	0.42857781	0.7083251	-0.27975	Arhgef7	NO	NO	NO	YES	NO	NO
chr3	144829575	144829660	86	6	0.53644558	0.87046698	-0.33402	Clca3b	NO	NO	NO	YES	NO	NO
chr9	56886773	56886876	104	9	0.52990236	0.7695336	-0.23963	Cspg4	NO	NO	YES	NO	NO	NO
chr17	24187559	24187653	95	5	0.38057961	0.53523303	-0.15465	Tbc1d24	NO	NO	NO	YES	NO	NO
chr12	33173896	33174074	179	5	0.26772662	0.74333301	-0.47561	Atxn7l1	NO	NO	NO	YES	NO	NO
chr7	45537749	45537807	59	5	0.75190581	0.6230593	0.128847	Plekha4	NO	NO	YES	NO	NO	NO
chr2	164080964	164081017	54	4	0.58254278	0.7779324	-0.19539	Stk4	NO	NO	NO	YES	NO	NO
chr4	59423948	59424032	85	5	0.17442484	0.73630024	-0.56188	Susd1	NO	NO	NO	YES	NO	NO
chr2	32454946	32455014	69	5	0.72073081	0.55616668	0.164564	Naif1	NO	NO	YES	NO	NO	NO
chr14	69859249	69859413	165	8	0.82241993	0.58705059	0.235369	Pebp4	NO	NO	NO	YES	NO	NO
chr4	137951842	137951907	66	5	0.04048691	0.6760722	-0.63559	Ece1	NO	NO	NO	YES	NO	NO
chr11	102665628	102665699	72	10	0.05294748	0.23059593	-0.17765	Gm1564	NO	YES	NO	NO	NO	NO
chr15	99816369	99816465	97	7	0.09115897	0.7343203	-0.64316	Lima1	NO	NO	NO	YES	NO	NO
chr15	78680459	78680548	90	5	0.87864788	0.48108832	0.39756	Elfn2	NO	NO	NO	YES	NO	NO
chr4	141845696	141845768	73	5	0.63907907	0.67376213	-0.03468	Ctrc	NO	NO	NO	YES	NO	NO
chr18	21197948	21198096	149	6	0.18099897	0.53299576	-0.352	Garem	NO	NO	NO	YES	NO	NO
chr11	116620167	116620227	61	5	0.35118611	0.44243892	-0.09125	Rhbdf2	NO	NO	NO	YES	NO	NO
chr14	54408530	54408668	139	11	0.4105136	0.64170448	-0.23119	Slc7a7	NO	NO	YES	NO	NO	NO
chr8	39544385	39544500	116	5	0.13604737	0.42505243	-0.28901	Mir669e	NO	NO	NO	YES	NO	NO
chr15	89288368	89288479	112	6	0.88951538	0.67344597	0.216069	Sbf1	NO	NO	NO	NO	YES	NO
chr2	20743565	20743737	173	11	0.44381203	0.71340561	-0.26959	Etl4	NO	NO	YES	NO	NO	NO
chr4	149634701	149634795	95	6	0.61057315	0.58222203	0.028351	Clstn1	NO	NO	YES	NO	NO	NO
chr11	78474886	78474946	61	4	0.23345024	0.67285218	-0.4394	Sarm1	NO	NO	YES	NO	NO	NO
chr14	66004899	66004983	85	7	0.37018858	0.28797033	0.082218	Gulo	NO	NO	NO	YES	NO	NO
chr17	48168090	48168168	79	9	0.79260948	0.6329462	0.159663	A530064D0	YES	NO	NO	NO	NO	NO
chr16	16508079	16508150	72	4	0.24040369	0.94154307	-0.70114	Fgd4	NO	NO	NO	YES	NO	NO

chr12	111789885	111789972	88	7	0.52485787	0.78222409	-0.25737	Klc1	NO	NO	NO	YES	NO	NO
chr12	34067277	34067333	57	5	0.7809427	0.53500391	0.245939	Hdac9	NO	NO	NO	YES	NO	NO
chr4	134243869	134243965	97	5	0.33779113	0.75431659	-0.41653	E130218I03	NO	YES	NO	NO	NO	NO
chr11	62664956	62665048	93	5	0.47968202	0.67071956	-0.19104	Mmgt2	NO	NO	YES	NO	NO	NO
chr3	153933092	153933208	117	9	0.56207763	0.77705555	-0.21498	Acadm	NO	NO	NO	YES	NO	NO
chr5	122756528	122756602	75	5	0.81700045	0.6920501	0.12495	Camkk2	NO	NO	NO	YES	NO	NO
chr8	95707300	95707475	176	9	0.32850882	0.71876246	-0.39025	Ndrg4	NO	NO	NO	YES	NO	NO
chr10	61523913	61523989	77	8	0.88329885	0.72660184	0.156697	Lrrc20	NO	NO	NO	YES	NO	NO
chr7	79386504	79386560	57	11	0.47056228	0.71135343	-0.24079	Rlbp1	NO	NO	NO	YES	NO	NO
chr4	24837170	24837271	102	7	0.19319146	0.67344701	-0.48026	Klhl32	NO	NO	NO	YES	NO	NO
chr4	141614624	141614771	148	6	0.87814667	0.73471897	0.143428	AI507597	NO	YES	NO	YES	NO	NO
chr14	69796364	69796448	85	8	0.9305178	0.66252075	0.267997	Rhobtb2	NO	NO	YES	NO	NO	NO
chr11	97146544	97146615	72	5	0.90531591	0.38546111	0.519855	Tbkbp1	NO	NO	YES	NO	NO	NO
chr17	26925287	26925419	133	8	0.12134288	0.67291134	-0.55157	Kifc5b	NO	NO	NO	YES	NO	NO
chr14	40844643	40844740	98	7	0.19223168	0.66844297	-0.47621	Sh2d4b	NO	NO	NO	YES	NO	NO
chr9	123454642	123454774	133	6	0.39389174	0.60484421	-0.21095	Lars2	NO	NO	NO	YES	NO	NO
chr4	133681655	133681740	86	7	0.45576078	0.67979972	-0.22404	Arid1a	NO	NO	YES	NO	NO	NO
chr11	81968002	81968060	59	4	0.27619722	0.63095119	-0.35475	Asic2	NO	NO	YES	NO	NO	NO
chr11	95299999	95300054	56	11	0.47845847	0.71638344	-0.23792	Kat7	NO	NO	YES	NO	NO	NO
chr11	117359503	117359577	75	8	0.5830716	0.63037374	-0.0473	9-Sep	NO	NO	NO	YES	NO	NO
chr3	142561348	142561434	87	4	0.93381312	0.68131668	0.252496	Gbp3	NO	NO	NO	YES	NO	NO
chr7	121487195	121487337	143	4	0.36877594	0.61635285	-0.24758	Hs3st2	NO	NO	NO	YES	NO	NO
chr15	4480642	4480726	85	4	0.20216591	0.70534476	-0.50318	Plcxd3	NO	NO	NO	YES	NO	NO
chr6	126533811	126533910	100	7	0.88486525	0.53519007	0.349675	Kcna5	NO	NO	YES	NO	NO	NO
chr17	27887081	27887155	75	9	0.51424201	0.67566851	-0.16143	Uhrf1bp1	NO	NO	YES	NO	NO	NO
chr2	76726166	76726246	81	10	0.67295424	0.75990418	-0.08695	Ttn	NO	NO	YES	NO	NO	NO
chr11	63964157	63964250	94	5	0.03157367	0.7350541	-0.70348	Cox10	NO	NO	YES	NO	NO	NO
chr4	152060504	152060566	63	7	0.45284517	0.74166248	-0.28882	Nol9	NO	NO	NO	YES	NO	YES
chr17	86642608	86642740	133	7	0.66657831	0.74944875	-0.08287	Prkce	NO	NO	NO	YES	NO	NO
chr11	55409832	55409917	86	5	0.9432205	0.72514648	0.218074	Sparc	NO	NO	NO	YES	NO	NO
chrX	134476387	134476496	110	7	0.27839259	0.69866188	-0.42027	Taf7l	NO	YES	YES	NO	NO	NO
chr5	96776678	96776738	61	5	0.37504654	0.63807182	-0.26303	Fras1	NO	NO	YES	NO	NO	NO
chr9	49710975	49711125	151	4	0.59253757	0.75517187	-0.16263	Ncam1	NO	NO	NO	YES	NO	NO
chr7	19451174	19451231	58	5	0.84693552	0.71756149	0.129374	Mark4	NO	NO	NO	YES	NO	NO
chr11	116077411	116077533	123	6	0.60149693	0.46794545	0.133551	Wbp2	NO	NO	NO	NO	NO	YES
chr11	115988949	115989033	85	8	0.18159295	0.64486081	-0.46327	Itgb4	NO	NO	YES	NO	NO	NO
chr4	133063289	133063376	88	5	0.29704024	0.81638879	-0.51935	Ahdc1	NO	NO	YES	NO	NO	NO
chr14	59573466	59577136	3671	4	0.20763711	0.72050744	-0.51287	Cdadc1	NO	NO	YES	YES	NO	NO

chrX	157568685	157568781	97	8	0.8203826	0.57295221	0.24743	Yy2	NO	NO	YES	NO	NO	NO
chr1	75186569	75186619	51	4	0.20313295	0.68334544	-0.48021	Atg9a	NO	NO	YES	NO	YES	NO
chr4	141614624	141614771	148	6	0.87814667	0.73471897	0.143428	Tmem82	NO	NO	YES	NO	NO	NO
chr11	107707819	107707930	112	6	0.17407676	0.37182245	-0.19775	Cacng1	NO	NO	NO	YES	NO	NO
chr11	63964157	63964250	94	5	0.03157367	0.7350541	-0.70348	9630013K1	YES	YES	NO	NO	NO	NO
chr2	60255801	60255893	93	5	0.30048414	0.61755184	-0.31707	Cd302	NO	NO	NO	YES	NO	NO
chr11	103015851	103015959	109	4	0.79008399	0.45650998	0.333574	Dcakd	NO	NO	NO	YES	NO	NO
chr10	78000791	78000848	58	6	0.53358315	0.79646159	-0.26288	Pfkl	NO	NO	YES	NO	NO	NO
chr1	171418160	171418309	150	6	0.32318658	0.57858059	-0.25539	Tstd1	YES	NO	NO	NO	NO	NO
chr9	98805480	98805533	54	6	0.26070976	0.5290629	-0.26835	E330023G0	NO	NO	NO	YES	NO	NO
chr5	135353389	135353493	105	4	0.03233409	0.32700338	-0.29467	Trim50	NO	NO	YES	NO	NO	NO
chr8	27121395	27121475	81	9	0.03786844	0.39508556	-0.35722	Adgra3	NO	NO	YES	NO	NO	NO
chr10	77900897	77900991	95	7	0.22625637	0.71795155	-0.4917	Lrrc3	NO	NO	YES	NO	NO	NO
chr2	32069284	32069348	65	4	0.15659309	0.37042032	-0.21383	Fam78a	NO	NO	YES	NO	NO	NO
chr5	50253063	50253158	96	6	0.37760392	0.82707197	-0.44947	Mir8117	NO	NO	NO	NO	NO	YES
chr15	27871063	27871225	163	12	0.35477267	0.70067478	-0.3459	Trio	NO	NO	NO	YES	NO	NO
chr15	88712543	88712657	115	9	0.70724725	0.67431738	0.03293	Brd1	NO	NO	NO	YES	NO	NO
chr14	40916749	40916899	151	13	0.33779179	0.66997109	-0.33218	Tspan14	NO	NO	YES	NO	NO	NO
chr10	61536236	61536306	71	7	0.32708555	0.50691459	-0.17983	Lrrc20	NO	NO	NO	YES	NO	NO
chr8	113638856	113638918	63	9	0.7244224	0.64192913	0.082493	Mon1b	NO	NO	YES	NO	NO	NO
chr6	85269112	85272646	3535	5	0.83855307	0.60403307	0.23452	Sfxn5	NO	NO	YES	YES	NO	NO
chr11	35475777	35475833	57	5	0.16908967	0.24108473	-0.072	Slit3	NO	NO	NO	YES	NO	NO
chr19	7096468	7096532	65	4	0.24577739	0.19288174	0.052896	Flrt1	NO	NO	YES	NO	NO	NO
chr11	69854162	69854237	76	4	0.02632113	0.31938907	-0.29307	Tnk1	NO	NO	NO	YES	NO	NO
chr5	122404516	122404587	72	5	0.83146599	0.74398128	0.087485	Arpc3	NO	NO	NO	YES	NO	NO
chr12	108648412	108648538	127	7	0.5138263	0.80036363	-0.28654	Evl	NO	NO	YES	NO	NO	NO
chr17	25959915	25959981	67	6	0.36553037	0.70878189	-0.34325	Capn15	NO	NO	YES	NO	NO	NO
chr5	112308196	112308290	95	8	0.19925167	0.72704544	-0.52779	Tpst2	NO	NO	YES	NO	NO	NO
chr13	42158094	42158151	58	5	0.39593932	0.77942246	-0.38348	Hivep1	NO	NO	YES	NO	NO	NO
chr9	15315937	15315990	54	6	0.82811022	0.72692741	0.101183	Cep295	NO	NO	NO	NO	NO	YES
chr4	5798813	5798920	108	7	0.22373929	0.6158217	-0.39208	Fam110b	NO	NO	YES	NO	NO	NO
chr4	134864580	134864703	124	4	0.22385714	0.50463829	-0.28078	Rhd	NO	NO	YES	NO	NO	NO
chr11	98821857	98821918	62	4	0.24531184	0.69109998	-0.44579	Casc3	NO	NO	NO	YES	NO	NO
chr6	96164781	96164867	87	6	0.15885734	0.4278757	-0.26902	Fam19a1	NO	NO	NO	YES	NO	NO
chr15	76713741	76713892	152	15	0.77736174	0.72310882	0.054253	Lrrc14	NO	NO	YES	NO	NO	NO
chr5	90215100	90215174	75	7	0.38455035	0.71961459	-0.33506	Cox18	NO	NO	YES	NO	YES	NO
chr1	72839485	72839555	71	4	0.24233119	0.21691437	0.025417	Igfbp2	NO	NO	NO	YES	NO	NO
chr14	27203441	27203502	62	5	0.83094273	0.55107284	0.27987	Arhgef3	NO	NO	NO	YES	NO	NO

chr15	76713741	76713892	152	15	0.77736174	0.72310882	0.054253	Lrrc24	NO	NO	NO	NO	NO	YES
chrX	163305795	163305884	90	11	0.54036438	0.64365733	-0.10329	Bc1	NO	NO	NO	YES	NO	NO
chr8	87923007	87923117	111	4	0.62849683	0.41543026	0.213067	Zfp423	NO	NO	NO	YES	NO	NO
chr7	16886259	16887626	1368	7	0.73338911	0.54418765	0.189201	Dact3	NO	NO	NO	NO	YES	NO
chr11	22973087	22973161	75	11	0.19354835	0.35044017	-0.15689	Zrsr1	NO	YES	NO	NO	NO	NO
chr5	101935507	101935572	66	7	0.43836974	0.71064513	-0.27228	Wdfy3	NO	NO	NO	YES	NO	NO
chr4	63153943	63154011	69	7	0.13210964	0.50245318	-0.37034	Ambp	NO	NO	YES	NO	NO	NO
chr9	58158108	58158218	111	4	0.81484912	0.53711726	0.277732	Islr	NO	NO	YES	NO	NO	NO
chr1	34291819	34291873	55	6	0.32776317	0.33152649	-0.00376	Dst	NO	NO	YES	NO	NO	NO
chr17	33932644	33932713	70	4	0.31762731	0.59706374	-0.27944	Rgl2	NO	NO	YES	NO	NO	NO
chr4	62312337	62312487	151	5	0.16875873	0.7085314	-0.53977	Fkbp15	NO	NO	NO	YES	NO	NO
chr7	28559646	28559748	103	15	0.27931888	0.63630175	-0.35698	Mir7049	NO	NO	NO	NO	NO	YES
chr2	177759514	177759612	99	7	0.42516496	0.23905484	0.18611	Gm14322	NO	NO	NO	YES	NO	NO
chr5	111286369	111286443	75	7	0.73668027	0.53466323	0.202017	Ttc28	NO	NO	YES	NO	NO	NO
chr11	53371393	53371512	120	5	0.24531184	0.76442941	-0.51912	Aff4	NO	NO	NO	YES	NO	NO
chr18	84729321	84729393	73	5	0.36560228	0.76312415	-0.39752	Fam69c	NO	NO	NO	YES	NO	NO
chr18	82673007	82673122	116	6	0.27828365	0.56043817	-0.28215	Zfp236	NO	NO	NO	YES	NO	NO
chr4	151983269	151983320	52	5	0.37599907	0.48921579	-0.11322	Thap3	NO	NO	YES	NO	NO	NO
chr17	33961045	33961168	124	11	0.65208851	0.71444242	-0.06235	Vps52	NO	NO	NO	YES	NO	NO
chr7	143475960	143476024	65	7	0.2527969	0.48594094	-0.23314	Slc22a18	NO	NO	NO	YES	NO	NO
chr10	120873300	120873372	73	4	0.0397888	0.26235685	-0.22257	Msrb3	NO	NO	NO	YES	NO	NO
chr5	113095509	113095562	54	5	0.48142567	0.63957736	-0.15815	2900026A0	NO	NO	NO	YES	NO	NO
chr14	35432905	35433004	100	9	0.42072834	0.82257823	-0.40185	Grid1	NO	NO	NO	YES	NO	NO
chr11	20021228	20021288	61	7	0.83703017	0.73006869	0.106961	Spred2	NO	NO	YES	NO	NO	NO
chrX	157568685	157568781	97	8	0.8203826	0.57295221	0.24743	Mbtps2	NO	NO	NO	YES	NO	NO
chr11	102149031	102149082	52	4	0.81187291	0.58828379	0.223589	Nags	NO	NO	YES	NO	NO	NO
chr17	47744039	47744135	97	5	0.70322518	0.73044332	-0.02722	Tfeb	NO	NO	NO	YES	NO	NO
chr2	164002436	164002489	54	5	0.52920639	0.72019252	-0.19099	Ywhab	NO	NO	NO	YES	NO	NO
chr12	73288437	73288487	51	5	0.46339863	0.75347168	-0.29007	Slc38a6	NO	NO	YES	NO	NO	NO
chr7	60005043	60005093	51	5	0.05695826	0.29948149	-0.24252	Snurf	NO	YES	YES	NO	NO	NO
chr16	30959937	30959995	59	8	0.44002275	0.73276965	-0.29275	Xxylt1	NO	NO	NO	YES	NO	NO
chr2	132142582	132142659	78	7	0.54422977	0.78334312	-0.23911	Slc23a2	NO	NO	NO	YES	NO	NO
chr18	59373777	59373840	64	11	0.53883696	0.75767638	-0.21884	Chsy3	NO	NO	NO	YES	NO	NO
chr5	137295953	137296013	61	5	0.38110494	0.70005757	-0.31895	Ache	NO	NO	NO	NO	NO	YES
chr7	127384998	127385102	105	8	0.77210698	0.77594433	-0.00384	9130019O2	NO	NO	YES	NO	NO	NO
chr12	108309496	108311872	2377	4	0.35928086	0.41838894	-0.05911	Hhipl1	NO	NO	NO	YES	NO	NO
chr18	32258217	32258296	80	9	0.78101232	0.58221219	0.1988	Ercc3	NO	NO	NO	YES	NO	NO
chr7	142652118	142652181	64	5	0.68160156	0.29848717	0.383114	Igf2	NO	NO	NO	NO	YES	NO

chr8	105913425	105913500	76	8	0.56440078	0.75819733	-0.1938	Pskh1	NO	NO	YES	NO	NO	NO
chr11	77471564	77471660	97	10	0.70267896	0.4560364	0.246643	Ankrd13b	NO	NO	YES	NO	NO	NO
chr4	130371459	130371556	98	5	0.2855058	0.70605429	-0.42055	Snrnp40	NO	NO	NO	YES	NO	NO
chr17	49458593	49458690	98	8	0.62639236	0.33577786	0.290615	Daam2	NO	NO	YES	NO	NO	NO
chr4	103026088	103026202	115	8	0.45239735	0.71299241	-0.2606	Insl5	NO	NO	NO	YES	NO	NO
chr18	34758117	34758179	63	5	0.45930486	0.63644948	-0.17714	Fam53c	YES	NO	NO	NO	NO	NO
chr15	79084085	79084143	59	5	0.42221242	0.7842192	-0.36201	Eif3l	NO	NO	YES	NO	NO	NO
chr8	47827890	47827951	62	8	0.42597849	0.63056896	-0.20459	Wwc2	NO	NO	NO	NO	YES	NO
chr2	25094900	25094950	51	7	0.42956746	0.25508476	0.174483	Noxa1	NO	YES	NO	NO	NO	NO
chr19	43868545	43868619	75	4	0.39225708	0.69061564	-0.29836	Dnmbp	NO	NO	NO	YES	NO	NO
chr9	119042684	119042788	105	11	0.48702823	0.76964885	-0.28262	Ctdspl	NO	NO	NO	NO	YES	NO
chr1	131225017	131225074	58	4	0.09632336	0.58057765	-0.48425	Rassf5	NO	NO	NO	YES	NO	NO
chr8	122336032	122336093	62	10	0.33673969	0.02618119	0.310558	Zfpm1	NO	NO	YES	NO	NO	NO
