# Supplementary material for: Intrauterine hyperglycemia exposure results in intergenerational inheritance via DNA methylation reprogramming on F1 PGCs
Source: Epigenetics Chromatin. 2018 May 25;11:20. doi: 10.1186/s13072-018-0192-2 (PMC5968593; doi:10.1186/s13072-018-0192-2)
Supplement: Supplementary file 5 — Additional file 5. IPA pathway analysis of RRBS methylome sequencing. [file 13072_2018_192_MOESM5_ESM.pdf]

Additional file 5

Table S3. Pathway analysis by IPA

Ingenuity Canonical Pathways	-log(p-value)	Ratio	Molecules
Semaphorin Signaling in	4.32	0.115	FYN,PAK4,SEMA4D,RHOJ,FNBP1,LIMK1
Axonal Guidance Signaling	3.74	0.036	SLIT3,KLC1,FYN,PAK4,ARHGEF7,SOS2,ADAMTS2,LIMK1,VEGFA,SEMA4D,AKT1,PRKCE,ARPC3,RASSF5,WNT5B,PRKCA
Integrin Signaling	3.42	0.0472	FYN,PAK4,AKT1,ARHGEF7,SOS2,ARPC3,RHOJ,ITGB4,TTN,FNBP1
RhoGDI Signaling	3.38	0.0511	PAK4,ARHGEF7,ARPC3,RHOJ,ARHGEF3,ESR1,FNBP1,LIMK1,PRKCA
Molecular Mechanisms of Cancer	3.35	0.0361	FYN,PAK4,ARHGEF7,SOS2,APAF1,RHOJ,TCF3,AKT1,PRKCE,ARHGEF3,FNBP1,WNT5B,CAMK2B,PRKCA
VEGF Family Ligand-Receptor Interactions	3.06	0.0682	VEGFA,AKT1,FLT4,SOS2,PRKCE,PRKCA
IL-8 Signaling	3.05	0.0459	VEGFA,AKT1,FLT4,PRKCE,RHOJ,IRAK4,FNBP1,LIMK1,PRKCA
Non-Small Cell Lung Cancer Signaling	2.53	0.0649	STK4,AKT1,SOS2,RASSF5,PRKCA
Phospholipase C Signaling	2.51	0.0383	HDAC9,FYN,ARHGEF7,SOS2,PRKCE,RHOJ,ARHGEF3,FNBP1,PRKCA
Glioma Signaling	2.5	0.0531	IGF2,AKT1,SOS2,PRKCE,PRKCA,CAMK2B
Prolactin Signaling	2.39	0.0602	FYN,SOS2,SOCS2,PRKCE,PRKCA
Natural Killer Cell Signaling	2.37	0.05	FYN,PAK4,AKT1,SOS2,PRKCE,PRKCA
Role of Tissue Factor in Cancer	2.37	0.05	VEGFA,FYN,AKT1,FGA,LIMK1,PRKCA
Glioblastoma Multiforme Signaling	2.35	0.0435	IGF2,AKT1,SOS2,RHOJ,TCF3,FNBP1,WNT5B
Regulation of Actin-based Motility by Rho	2.33	0.0581	PAK4,ARPC3,RHOJ,FNBP1,LIMK1
eNOS Signaling	2.29	0.0424	VEGFA,AKT1,FLT4,PRKCE,ESR1,HSPA1L,PRKCA
HER-2 Signaling in Breast Cancer	2.29	0.0568	AKT1,SOS2,PRKCE,ITGB4,PRKCA
Aldosterone Signaling in Epithelial Cells	2.27	0.0419	ASIC2,SOS2,HSPA13,PRKCE,HSPA1L,DNAJC11,PRKCA
Factors Promoting Cardiogenesis in Vertebrates	2.26	0.0562	ACVR1,PRKCE,TCF7L1,TCF3,PRKCA
Actin Nucleation by ARP-WASP Complex	2.26	0.0714	SOS2,ARPC3,RHOJ,FNBP1
Acute Phase Response	2.24	0.0414	AKT1,AMBP,SOS2,SOCS2,SERPINE1,FGA,TCF3
Fcγ Receptor-mediated Phagocytosis in Macrophages and Monocytes	2.18	0.0538	FYN,AKT1,ARPC3,PRKCE,PRKCA
Ephrin Receptor Signaling	2.18	0.0405	VEGFA,FYN,PAK4,AKT1,SOS2,ARPC3,LIMK1
Actin Cytoskeleton Signaling	2.14	0.0362	PAK4,ARHGEF7,SOS2,MYH3,TRIO,ARPC3,TTN,LIMK1
Inhibition of Angiogenesis by TSP1	2.12	0.0938	VEGFA,FYN,AKT1
ErbB Signaling	2.11	0.0515	PAK4,AKT1,SOS2,PRKCE,PRKCA
FAK Signaling	2.09	0.051	FYN,PAK4,AKT1,ARHGEF7,SOS2
VEGF Signaling	2.05	0.05	VEGFA,AKT1,FLT4,SOS2,PRKCA

Cholecystokinin/Gastrin-mediated Signaling	2.04	0.0495	SOS2,PRKCE,RHOJ,FNBP1,PRKCA
Epithelial Adherens Junction Signaling	2.01	0.042	AKT1,ACVR1,MYH3,ARPC3,TCF7L1,TCF3
IGF-1 Signaling	1.95	0.0472	AKT1,YWHAB,SOS2,SOCS2,IGFBP2
ILK Signaling	1.94	0.0363	VEGFA,AKT1,SH2B2,MYH3,RHOJ,ITGB4,FNBP1
Myc Mediated Apoptosis Signaling	1.92	0.0571	AKT1,YWHAB,SOS2,APAF1
ErbB4 Signaling	1.92	0.0571	AKT1,SOS2,PRKCE,PRKCA
Nitric Oxide Signaling in the Cardiovascular System	1.92	0.0463	VEGFA,AKT1,FLT4,PRKCE,PRKCA
Colorectal Cancer Metastasis Signaling	1.91	0.0329	VEGFA,AKT1,SOS2,RHOJ,TCF7L1,TCF3,FNBP1,WNT5B
ERK/MAPK Signaling	1.87	0.0352	FYN,PAK4,YWHAB,SOS2,PRKCE,ESR1,PRKCA
Thrombin Signaling	1.85	0.0348	AKT1,PRKCE,RHOJ,ARHGEF3,FNBP1,PRKCA,CAMK2B
Signaling by Rho Family GTPases	1.83	0.0319	PAK4,SEPT9,ARHGEF7,ARPC3,RHOJ,ARHGEF3,FNBP1,LIMK1
Mechanisms of Viral Exit from Host Cells	1.83	0.0732	PRKCE,LMNB1,PRKCA
Role of Macrophages, Fibroblasts and Endothelial Cells in Rheumatoid Arthritis	1.82	0.0297	VEGFA,AKT1,PRKCE,TCF7L1,TCF3,IRAK4,WNT5B,PRKCA,CAMK2B
Breast Cancer Regulation by Stathmin1	1.82	0.0343	ARHGEF7,SOS2,PRKCE,ARHGEF3,LIMK1,PRKCA,CAMK2B
Granzyme B Signaling	1.77	0.125	APAF1,LMNB1
Fc Epsilon RI Signaling	1.75	0.042	FYN,AKT1,SOS2,PRKCE,PRKCA
CXCR4 Signaling	1.74	0.0366	PAK4,AKT1,PRKCE,RHOJ,FNBP1,PRKCA
Erythropoietin Signaling	1.71	0.0494	AKT1,SOS2,PRKCE,PRKCA
Renal Cell Carcinoma Signaling	1.71	0.0494	VEGFA,PAK4,AKT1,SOS2
Tec Kinase Signaling	1.7	0.0357	FYN,PAK4,PRKCE,RHOJ,FNBP1,PRKCA
Type II Diabetes Mellitus Signaling	1.66	0.0397	AKT1,SH2B2,SOCS2,PRKCE,PRKCA
PI3K Signaling in B	1.66	0.0397	FYN,AKT1,SH2B2,PLEKHA4,CAMK2B
Growth Hormone Signaling	1.64	0.0471	IGF2,SOCS2,PRKCE,PRKCA
Estrogen Receptor Signaling	1.63	0.0391	ERCC3,TRRAP,SOS2,ESR1,TAF7L
Neuregulin Signaling	1.63	0.0465	AKT1,SOS2,PRKCE,PRKCA
TGF-β Signaling	1.61	0.046	ZNF423,SOS2,ACVR1,SERPINE1
G Beta Gamma Signaling	1.59	0.0455	AKT1,SOS2,PRKCE,PRKCA
HMGB1 Signaling	1.59	0.0382	KAT7,AKT1,RHOJ,SERPINE1,FNBP1
p70S6K Signaling	1.59	0.0382	AKT1,YWHAB,SOS2,PRKCE,PRKCA
GP6 Signaling Pathway	1.59	0.0382	FYN,AKT1,PRKCE,FGA,PRKCA
Adipogenesis pathway	1.58	0.0379	HDAC9,ZNF423,KAT7,AKT1,ERCC3
Glucocorticoid Receptor Signaling	1.56	0.0268	ARID1A,AKT1,ERCC3,SOS2,SERPINE1,ESR1,TAF7L,HSPA1L,KRT4
Hepatic Fibrosis / Hepatic Stellate Cell Activation	1.54	0.0328	VEGFA,IGF2,FLT4,MYH3,ECE1,SERPINE1
Acute Myeloid Leukemia Signaling	1.53	0.0435	AKT1,SOS2,TCF7L1,TCF3
Insulin Receptor Signaling	1.52	0.0365	FYN,ASIC2,AKT1,SH2B2,SOS2
RAR Activation	1.5	0.0321	VEGFA,ARID1A,AKT1,ERCC3,PRKCE,PRKCA

Regulation of the Epithelial-Mesenchymal Transition	1.5	0.0321	JAG2,AKT1,SOS2,TCF7L1,TCF3,WNT5B
Human Embryonic Stem Cell Pluripotency	1.48	0.0357	AKT1,ACVR1,TCF7L1,TCF3,WNT5B
GNRH Signaling	1.47	0.0355	PAK4,SOS2,PRKCE,PRKCA,CAMK2B
Role of NFAT in Cardiac Hypertrophy	1.47	0.0316	HDAC9,AKT1,SOS2,PRKCE,PRKCA,CAMK2B
Ovarian Cancer Signaling	1.45	0.035	VEGFA,AKT1,TCF7L1,TCF3,WNT5B
Glycerol-3-phosphate Shuttle	1.43	0.333	GPD1L
Huntington's Disease Signaling	1.42	0.0283	HDAC9,AKT1,SOS2,APAF1,PRKCE,HSPA1L,PRKC
PAK Signaling	1.42	0.04	PAK4,ARHGEF7,SOS2,LIMK1
mTOR Signaling	1.4	0.0305	VEGFA,AKT1,PRKCE,RHOJ,FNBP1,PRKCA
Virus Entry via Endocytic Pathways	1.39	0.0392	FYN,PRKCE,ITGB4,PRKCA
Wnt/Ca+ pathway	1.36	0.0484	PDE6C,WNT5B,PRKCA
Mouse Embryonic Stem Cell Pluripotency	1.35	0.0381	AKT1,SOS2,TCF7L1,TCF3
Geranylgeranyldiphosphate Biosynthesis	1.3	0.25	COX10
Androgen Signaling	1.29	0.0364	KAT7,ERCC3,PRKCE,PRKCA
Paxillin Signaling	1.29	0.0364	PAK4,ARHGEF7,SOS2,ITGB4
UVB-Induced MAPK Signaling	1.29	0.0455	AKT1,PRKCE,PRKCA
Gαq Signaling	1.29	0.0314	AKT1,PRKCE,RHOJ,FNBP1,PRKCA
p53 Signaling	1.28	0.036	HDAC9,WT1,AKT1,APAF1
EGF Signaling	1.26	0.0441	AKT1,SOS2,PRKCA
Chemokine Signaling	1.26	0.0441	LIMK1,PRKCA,CAMK2B
HGF Signaling	1.25	0.0351	AKT1,SOS2,PRKCE,PRKCA
Basal Cell Carcinoma Signaling	1.24	0.0435	TCF7L1,TCF3,WNT5B
Melatonin Signaling	1.23	0.0429	PRKCE,PRKCA,CAMK2B
G-Protein Coupled Receptor Signaling	1.22	0.0255	FYN,AKT1,SOS2,PDE6C,PRKCE,PRKCA,CAMK2B
Caveolar-mediated Endocytosis Signaling	1.21	0.0423	FYN,ITGB4,PRKCA
Role of JAK2 in Hormone-like Cytokine Signaling	1.21	0.0625	SH2B2,SOCS2
Germ Cell-Sertoli Cell Junction Signaling	1.2	0.0296	PAK4,AKT1,RHOJ,FNBP1,LIMK1
Wnt/β-catenin Signaling	1.2	0.0296	AKT1,ACVR1,TCF7L1,TCF3,WNT5B
PTEN Signaling	1.19	0.0336	MAST2,AKT1,FLT4,SOS2
Role of Wnt/GSK-3β Signaling in the Pathogenesis of Influenza	1.19	0.0411	TCF7L1,TCF3,WNT5B
GM-CSF Signaling	1.19	0.0411	AKT1,SOS2,CAMK2B
Role of NANOG in Mammalian Embryonic Stem Cell Pluripotency	1.18	0.0333	AKT1,SOS2,TCF7L1,WNT5B
Renin-Angiotensin Signaling	1.17	0.0331	PAK4,SOS2,PRKCE,PRKCA
RhoA Signaling	1.16	0.0328	SEPT9,ARPC3,TTN,LIMK1
Phagosome Formation	1.15	0.0325	PRKCE,RHOJ,FNBP1,PRKCA
Coagulation System	1.14	0.0571	SERPINE1,FGA
Chondroitin and Dermatan Biosynthesis	1.13	0.167	CHSY3
Zymosterol Biosynthesis	1.13	0.167	LBR

VDR/RXR Activation	1.13	0.039	WT1,PRKCE,PRKCA
FXR/RXR Activation	1.13	0.032	LIPC,AKT1,AMBP,FGA
CCR3 Signaling in Eosinophils	1.12	0.0317	PAK4,PRKCE,LIMK1,PRKCA
Cdc42 Signaling	1.09	0.031	PAK4,CDC42BPA,ARPC3,LIMK1
14-3-3-mediated Signaling	1.08	0.0308	AKT1,YWHAB,PRKCE,PRKCA
Macropinocytosis Signaling	1.08	0.037	PRKCE,ITGB4,PRKCA
CREB Signaling in Neurons	1.07	0.0272	AKT1,SOS2,PRKCE,PRKCA,CAMK2B
Thyroid Cancer Signaling	1.06	0.0513	TCF7L1,TCF3
IL-3 Signaling	1.06	0.0361	AKT1,PRKCE,PRKCA
JAK/Stat Signaling	1.06	0.0361	AKT1,SOS2,SOCS2
B Cell Receptor Signaling	1.04	0.0266	AKT1,SOS2,RASSF5,TCF3,CAMK2B
Role of PKR in Interferon Induction and Antiviral Response	1.02	0.0488	AKT1,APAF1
IL-7 Signaling Pathway	1.02	0.0349	FYN,AKT1,SOS2
NF-κB Activation by Viruses	1.01	0.0345	AKT1,PRKCE,PRKCA
Production of Nitric Oxide and Reactive Oxygen Species in Macrophages	1.01	0.0259	AKT1,PRKCE,RHOJ,FNBP1,PRKCA
UVC-Induced MAPK Signaling	0.989	0.0465	PRKCE,PRKCA
Apoptosis Signaling	0.987	0.0337	APAF1,PRKCE,PRKCA
Leucine Degradation I	0.966	0.111	ACADM
FGF Signaling	0.965	0.033	AKT1,SOS2,PRKCA
Chondroitin Sulfate Biosynthesis (Late Stages)	0.956	0.0444	CHSY3,HS3ST2
Reelin Signaling in Neurons	0.954	0.0326	FYN,AKT1,ARHGEF3
nNOS Signaling in Neurons	0.94	0.0435	PRKCE,PRKCA
Protein Kinase A Signaling	0.939	0.0208	YWHAB,PDE6C,PRKCE,TCF7L1,TCF3,TTN,PRKCA ,CAMK2B
Assembly of RNA Polymerase I Complex	0.923	0.1	POLR1B
Oleate Biosynthesis II (Animals)	0.923	0.1	UFSP1
TNFR1 Signaling	0.91	0.0417	PAK4,APAF1
TR/RXR Activation	0.894	0.0306	AKT1,UCP2,FGA
Assembly of RNA Polymerase II Complex	0.881	0.04	ERCC3,TAF7L
Amyloid Processing	0.881	0.04	AKT1,PRKCE
Docosahexaenoic Acid (DHA) Signaling	0.853	0.0385	AKT1,APAF1
Hepatic Cholestasis	0.852	0.0253	PRKCE,ESR1,IRAK4,PRKCA
Nur77 Signaling in T Lymphocytes	0.84	0.0377	HDAC9,APAF1
Chondroitin Sulfate Biosynthesis	0.84	0.0377	CHSY3,HS3ST2
Chronic Myeloid Leukemia Signaling	0.83	0.0286	HDAC9,AKT1,SOS2
Transcriptional Regulatory Network in Embryonic Stem	0.827	0.037	TCF7L1,ZFHX3
Unfolded protein response	0.827	0.037	HSPA1L,MBTPS2
autophagy	0.827	0.037	WDFY3,ATG9A
Cholesterol Biosynthesis I	0.817	0.0769	LBR
Cholesterol Biosynthesis II (via 24,25-dihydrolanosterol)	0.817	0.0769	LBR

Cholesterol Biosynthesis III (via Desmosterol)	0.817	0.0769	LBR
Dermatan Sulfate Biosynthesis	0.814	0.0364	CHSY3,HS3ST2
Telomerase Signaling	0.805	0.0278	HDAC9,AKT1,SOS2
Corticotropin Releasing Hormone Signaling	0.796	0.0275	VEGFA,PRKCE,PRKCA
Phenylalanine Degradation IV (Mammalian, via Side Chain)	0.787	0.0714	ACSF3
Telomere Extension by Telomerase	0.76	0.0667	PINX1
Vitamin-C Transport	0.76	0.0667	SLC23A2
Calcium Signaling	0.76	0.0233	HDAC9,MYH3,CAMKK2,CAMK2B
Neuropathic Pain Signaling In Dorsal Horn Neurons	0.756	0.0263	PRKCE,PRKCA,CAMK2B
Calcium-induced T Lymphocyte Apoptosis	0.755	0.0333	PRKCE,PRKCA
Ephrin A Signaling	0.755	0.0333	FYN,LIMK1
Rac Signaling	0.741	0.0259	PAK4,ARPC3,LIMK1
Extrinsic Prothrombin Activation Pathway	0.735	0.0625	FGA
Superpathway of Geranylgeranyldiphosphate Biosynthesis I (via Mevalonate)	0.735	0.0625	COX10
Parkinson's Signaling	0.735	0.0625	PRKN
iCOS-iCOSL Signaling in T Helper Cells	0.726	0.0254	AKT1,PLEKHA4,CAMK2B
Synaptic Long Term Potentiation	0.719	0.0252	PRKCE,PRKCA,CAMK2B
Endometrial Cancer Signaling	0.712	0.0312	AKT1,SOS2
Pyridoxal 5'-phosphate Salvage Pathway	0.712	0.0312	PRKCE,LIMK1
IL-2 Signaling	0.712	0.0312	AKT1,SOS2
NGF Signaling	0.712	0.025	AKT1,SOS2,TRIO
Thrombopoietin Signaling	0.702	0.0308	PRKCE,PRKCA
fMLP Signaling in Neutrophils	0.697	0.0246	ARPC3,PRKCE,PRKCA
ERK5 Signaling	0.692	0.0303	AKT1,YWHAB
PI3K/AKT Signaling	0.69	0.0244	AKT1,YWHAB,SOS2
Sphingosine-1-phosphate Signaling	0.684	0.0242	AKT1,RHOJ,FNBP1
CCR5 Signaling in Macrophages	0.682	0.0299	PRKCE,PRKCA
Lymphotoxin β Receptor Signaling	0.682	0.0299	AKT1,APAF1
Agrin Interactions at Neuromuscular Junction	0.682	0.0299	PAK4,ARHGEF7
ErbB2-ErbB3 Signaling	0.673	0.0294	AKT1,SOS2
The Visual Cycle	0.668	0.0526	RLBP1
DNA damage-induced 14-3-3σ Signaling	0.668	0.0526	AKT1
Role of JAK1 and JAK3 in γc Cytokine Signaling	0.664	0.029	IL21,SH2B2
CD28 Signaling in T Helper Cells	0.663	0.0236	FYN,AKT1,ARPC3
NRF2-mediated Oxidative Stress Response	0.657	0.0211	AKT1,PRKCE,DNAJC11,PRKCA

PKCθ Signaling in T Lymphocytes	0.657	0.0234	FYN,SOS2,CAMK2B
IL-6 Signaling	0.657	0.0234	VEGFA,AKT1,SOS2
Glioma Invasiveness Signaling	0.654	0.0286	RHOJ,FNBP1
Role of MAPK Signaling in the Pathogenesis of Influenza	0.654	0.0286	AKT1,PRKCA
Gap Junction Signaling	0.652	0.0209	AKT1,SOS2,PRKCE,PRKCA
Endoplasmic Reticulum Stress Pathway	0.63	0.0476	MBTPS2
Hypoxia Signaling in the Cardiovascular System	0.628	0.0274	VEGFA,AKT1
P2Y Purigenic Receptor Signaling Pathway	0.625	0.0226	AKT1,PRKCE,PRKCA
STAT3 Pathway	0.619	0.027	FLT4,SOCS2
Protein Ubiquitination Pathway	0.615	0.0189	HSPA13,PRKN,USP34,HSPA1L,DNAJC11
Angiopoietin Signaling	0.594	0.026	PAK4,AKT1
Neurotrophin/TRK Signaling	0.594	0.026	AKT1,SOS2
Estrogen-Dependent Breast Cancer Signaling	0.587	0.0256	AKT1,ESR1
IL-22 Signaling	0.58	0.0417	AKT1
Tumoricidal Function of Hepatic Natural Killer Cells	0.58	0.0417	APAF1
Glycolysis I	0.58	0.0417	PFKL
Regulation of IL-2 Expression in Activated and Anergic T Lymphocytes	0.579	0.0253	FYN,SOS2
Hereditary Breast Cancer Signaling	0.578	0.0213	HDAC9,ARID1A,AKT1
IL-12 Signaling and Production in Macrophages	0.562	0.0208	AKT1,PRKCE,PRKCA
Estrogen-mediated S-phase Entry	0.55	0.0385	ESR1
FLT3 Signaling in Hematopoietic Progenitor Cells	0.549	0.0241	AKT1,SOS2
AMPK Signaling	0.539	0.0186	ARID1A,AKT1,PFKL,CAMKK2
Superpathway of Cholesterol Biosynthesis	0.536	0.037	LBR
α-Adrenergic Signaling	0.535	0.0235	PRKCE,PRKCA
Small Cell Lung Cancer	0.535	0.0235	AKT1,APAF1
GPCR-Mediated Nutrient Sensing in Enteroendocrine	0.535	0.0235	PRKCE,PRKCA
Systemic Lupus Erythematosus Signaling	0.53	0.0184	SNRPN,AKT1,SOS2,SNRNP40
HIPPO signaling	0.528	0.0233	STK4,YWHAB
IL-4 Signaling	0.528	0.0233	AKT1,SOS2
LPS-stimulated MAPK Signaling	0.521	0.023	PRKCE,PRKCA
PEDF Signaling	0.521	0.023	AKT1,TCF7L1
Relaxin Signaling	0.51	0.0195	VEGFA,AKT1,PDE6C
PDGF Signaling	0.501	0.0222	SOS2,PRKCA
Fatty Acid β-oxidation I	0.498	0.0333	ACADM
Death Receptor Signaling	0.489	0.0217	APAF1,LIMK1

Role of Osteoblasts, Osteoclasts and Chondrocytes in Rheumatoid Arthritis	0.487	0.0175	AKT1,TCF7L1,TCF3,WNT5B
Cytotoxic T Lymphocyte-mediated Apoptosis of Target Cells	0.487	0.0323	APAF1
Dopamine-DARPP32 Feedback in cAMP Signaling	0.482	0.0188	PRKCE,CAMKK2,PRKCA
Salvage Pathways of Pyrimidine Ribonucleotides	0.477	0.0213	PRKCE,LIMK1
Prostate Cancer Signaling	0.471	0.0211	AKT1,SOS2
Sumoylation Pathway	0.465	0.0208	RHOJ,FNBP1
Mitochondrial Dysfunction	0.46	0.0182	UCP2,COX10,PRKN
TWEAK Signaling	0.454	0.0294	APAF1
CTLA4 Signaling in Cytotoxic T Lymphocytes	0.453	0.0204	FYN,AKT1
Melanocyte Development and Pigmentation Signaling	0.453	0.0204	SH2B2,SOS2
PPARα/RXRα Activation	0.447	0.0179	SOS2,ACVR1,PRKCA
Nucleotide Excision Repair Pathway	0.444	0.0286	ERCC3
Notch Signaling	0.425	0.027	JAG2
tRNA Charging	0.416	0.0263	LARS2
Retinol Biosynthesis	0.416	0.0263	LIPC
NF-κB Signaling	0.407	0.0169	AKT1,FLT4,IRAK4
Type I Diabetes Mellitus Signaling	0.406	0.0187	APAF1,SOCS2
T Cell Receptor Signaling	0.401	0.0185	FYN,SOS2
Triacylglycerol Degradation	0.399	0.025	LIPC
Role of NFAT in Regulation of the Immune Response	0.396	0.0166	FYN,AKT1,SOS2
Amyotrophic Lateral Sclerosis Signaling	0.391	0.0182	VEGFA,APAF1
Intrinsic Prothrombin Activation Pathway	0.391	0.0244	FGA
tRNA Splicing	0.391	0.0244	PDE6C
Dermatan Sulfate Biosynthesis (Late Stages)	0.383	0.0238	HS3ST2
Endothelin-1 Signaling	0.378	0.0161	PRKCE,ECE1,PRKCA
iNOS Signaling	0.367	0.0227	IRAK4
HIF1α Signaling	0.364	0.0172	VEGFA,AKT1
IL-9 Signaling	0.36	0.0222	SOCS2
Pancreatic Adenocarcinoma Signaling	0.347	0.0167	VEGFA,AKT1
Sperm Motility	0.347	0.0167	PRKCE,PRKCA
LXR/RXR Activation	0.343	0.0165	AMBP,FGA
Xenobiotic Metabolism Signaling	0.343	0.0147	HS3ST2,PRKCE,PRKCA,CAMK2B
Clathrin-mediated Endocytosis Signaling	0.339	0.0152	VEGFA,ARPC3,ITGB4
Cell Cycle: G2/M DNA Damage Checkpoint Regulation	0.333	0.0204	YWHAB

Cancer Drug Resistance By Drug Efflux	0.333	0.0204	AKT1
Gustation Pathway	0.331	0.0161	ASIC2,PDE6C
Cellular Effects of Sildenafil (Viagra)	0.324	0.0159	CACNG1,MYH3
Phototransduction Pathway	0.321	0.0196	PDE6C
Sirtuin Signaling Pathway	0.32	0.0142	AKT1,UCP2,ATG9A,POLR1B
Leukocyte Extravasation Signaling	0.315	0.0146	PRKCE,RASSF5,PRKCA
CD27 Signaling in Lymphocytes	0.315	0.0192	APAF1
Role of Cytokines in Mediating Communication between Immune Cells	0.315	0.0192	IL21
Osteoarthritis Pathway	0.315	0.0146	VEGFA,TCF7L1,TCF3
FcγRIIB Signaling in B Lymphocytes	0.309	0.0189	AKT1
Role of Pattern Recognition Receptors in Recognition of Bacteria and Viruses	0.305	0.0153	PRKCE,PRKCA
EIF2 Signaling	0.298	0.0142	VEGFA,AKT1,SOS2
Melanoma Signaling	0.298	0.0182	AKT1
Aryl Hydrocarbon Receptor Signaling	0.288	0.0147	APAF1,ESR1
Heparan Sulfate Biosynthesis (Late Stages)	0.282	0.0172	HS3ST2
Phagosome Maturation	0.282	0.0145	DYNC1LI1,DYNC1I1
Retinoic acid Mediated Apoptosis Signaling	0.277	0.0169	APAF1
Phospholipases	0.277	0.0169	LIPC
Induction of Apoptosis by HIV1	0.272	0.0167	APAF1
CNTF Signaling	0.272	0.0167	AKT1
PCP pathway	0.272	0.0167	WNT5B
Synaptic Long Term Depression	0.269	0.0141	PRKCE,PRKCA
PXR/RXR Activation	0.258	0.0159	AKT1
Th2 Pathway	0.257	0.0137	JAG2,ACVR1
Heparan Sulfate Biosynthesis	0.249	0.0154	HS3ST2
Cell Cycle: G1/S Checkpoint Regulation	0.249	0.0154	HDAC9
Cardiac Hypertrophy Signaling	0.246	0.0129	AKT1,RHOJ,FNBP1
Remodeling of Epithelial Adherens Junctions	0.245	0.0152	ARPC3
Regulation of eIF4 and p70S6K Signaling	0.235	0.013	AKT1,SOS2
T Helper Cell Differentiation	0.233	0.0145	IL21
TREM1 Signaling	0.229	0.0143	AKT1
Ephrin B Signaling	0.218	0.0137	LIMK1
BMP signaling pathway	0.214	0.0135	ZNF423
Toll-like Receptor Signaling	0.211	0.0133	IRAK4
IL-15 Signaling	0.207	0.0132	AKT1
Role of PI3K/AKT Signaling in the Pathogenesis of Influenza	0.207	0.0132	AKT1

GDNF Family Ligand-Receptor Interactions	0.207	0.0132	SOS2
IL-17A Signaling in Airway Cells	0.201	0.0128	AKT1
Cyclins and Cell Cycle	0.197	0.0127	HDAC9
