# Supplementary material for: Intrauterine hyperglycemia exposure results in intergenerational inheritance via DNA methylation reprogramming on F1 PGCs
Source: Epigenetics Chromatin. 2018 May 25;11:20. doi: 10.1186/s13072-018-0192-2 (PMC5968593; doi:10.1186/s13072-018-0192-2)
Supplement: Supplementary file 6 — Additional file 6. DNA methylation validation in control and STZ-treated nondiabetic mice. [file 13072_2018_192_MOESM6_ESM.pdf]

**Additional file 6**

**Streptozotocin (STZ)-treated nondiabetic mice.** As described in materials and methods, pregnant mice in GDM group were treated with a single intraperitoneal injection of STZ to set up a GDM mice model. However, some mice did not developed diabetes after STZ injection. These mice with a consistent glucose level less than 5 mM were considered as an STZ-treated nondiabetic group (STZ group). We collected D13.5 male PGCs from STZ group, and conducted *Fyn* pyrosequencing to assess whether STZ has an impaction on DNA methylation (Fig. S4).

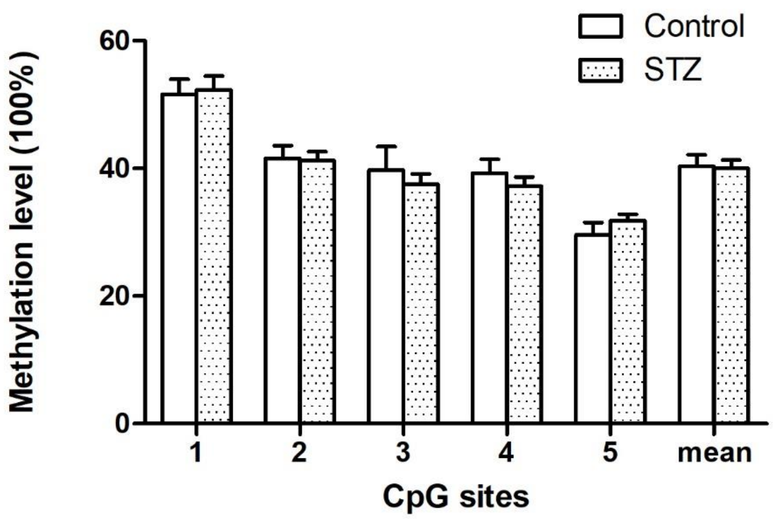


**Figure S6. Targeted bisulfite pyrosequencing-based DNA methylation validation of *Fyn* in control and STZ group male PGCs.** Methylation differences in D13.5 PGCs from control and STZ groups male fetuses (n=5 pregnant mice per group).
