# Supplementary material for: Intrauterine hyperglycemia exposure results in intergenerational inheritance via DNA methylation reprogramming on F1 PGCs
Source: Epigenetics Chromatin. 2018 May 25;11:20. doi: 10.1186/s13072-018-0192-2 (PMC5968593; doi:10.1186/s13072-018-0192-2)
Supplement: Supplementary file 7 — Additional file 7. Schematic diagram of the study [file 13072_2018_192_MOESM7_ESM.pdf]

Additional file 7

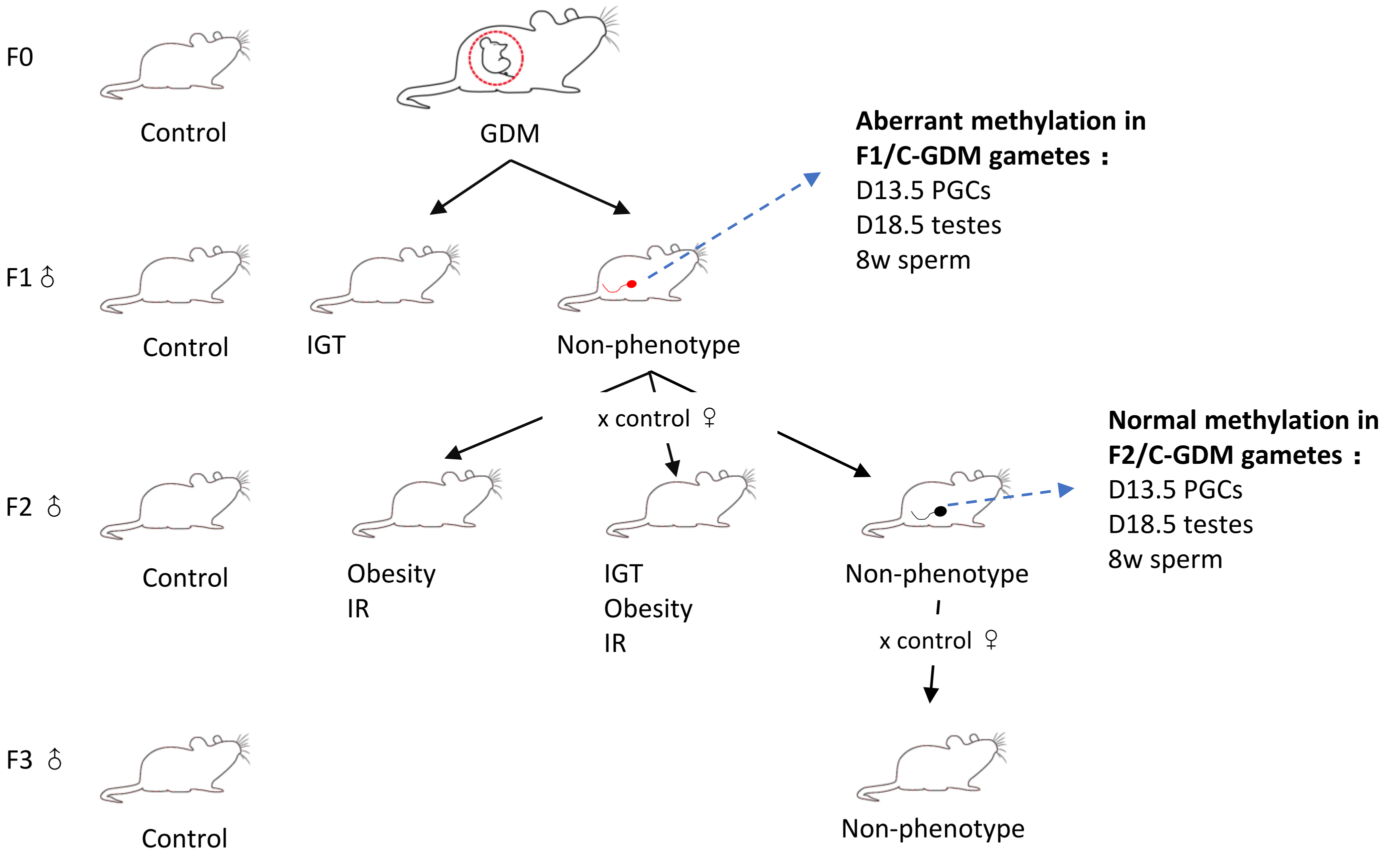

Figure S7. Experimental schematic. F1 offspring of GDM mice exhibited impaired glucose tolerance (IGT) or nondiabetic phenotypes. F2 offspring from nondiabetic F1-GDM father exhibited insulin resistance (IR), obesity and/or IGT diabetic phenotypes, while some showed nondiabetic phenotypes. F3 offspring from nondiabetic F2-GDM father exhibited nondiabetic phenotypes. Primordial germ cells (PGCs) in F1-GDM offspring exhibited aberrant DNA methylation status, and this epigenetic memory was inherited by somatic cells but not germ cells of F2 offspring.
